# Nanotwin-governed toughening mechanism in hierarchically structured biological materials

**DOI:** 10.1038/ncomms10772

**Published:** 2016-02-17

**Authors:** Yoon Ah Shin, Sheng Yin, Xiaoyan Li, Subin Lee, Sungmin Moon, Jiwon Jeong, Minhyug Kwon, Seung Jo Yoo, Young-Min Kim, Teng Zhang, Huajian Gao, Sang Ho Oh

**Affiliations:** 1 Department of Materials Science and Engineering, Pohang University of Science and Technology (POSTECH), Pohang 37673, Republic of Korea; 2 School of Engineering, Brown University, Providence, Rhode Island 02912, USA; 3 Center for Advanced Mechanics and Materials, Applied Mechanics Laboratory, Department of Engineering Mechanics, Tsinghua University, Beijing 100084, China; 4 IBS Center for Integrated Nanostructure Physics (CINAP), Institute for Basic Science, Sungkyunkwan University, Suwon 16419, Republic of Korea; 5 Nano-Bio Electron Microscopy Research Group, Korea Basic Science Institute (KBSI), Daejeon 34133, Republic of Korea; 6 Department of Energy Science, Sungkyunkwan University, Suwon 16419, Republic of Korea

## Abstract

As a natural biocomposite, *Strombus gigas*, commonly known as the giant pink queen conch shell, exhibits outstanding mechanical properties, especially a high fracture toughness. It is known that the basic building block of conch shell contains a high density of growth twins with average thickness of several nanometres, but their effects on the mechanical properties of the shell remain mysterious. Here we reveal a toughening mechanism governed by nanoscale twins in the conch shell. A combination of *in situ* fracture experiments inside a transmission electron microscope, large-scale atomistic simulations and finite element modelling show that the twin boundaries can effectively block crack propagation by inducing phase transformation and delocalization of deformation around the crack tip. This mechanism leads to an increase in fracture energy of the basic building block by one order of magnitude, and contributes significantly to that of the overall structure via structural hierarchy.

## Abstract (web summary)

As a natural biocomposite, *Strombus gigas*, commonly known as the giant pink queen conch shell, exhibits outstanding mechanical properties such as fracture toughness. Here, the authors show that these properties can be partially attributed to nanoscale twin boundaries in the basic building block of the shell.

## Graphical abstract

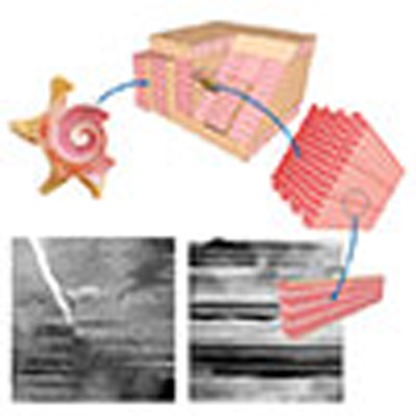

As an important class of natural biocomposite materials, mollusk shells possess remarkable mechanical strength and toughness as a consequence of their hierarchical structuring of soft organic and hard mineral constituents through biomineralization^1^,^2^,^3^,^4^,^5^,^6^,^7^. The *Strombus gigas* conch shell contains a high density of nanoscale {110} growth twins in its third-order aragonite lamellae, the basic building block of the material. Although the existence of nanotwinned aragonite has been known for decades^8^,^9^,^10^, its roles and functions in mechanical behaviours and properties of biological materials have received little attention, in spite of worldwide interests in biomimetic materials and numerous studies in recent years aimed to investigate the relationship between mechanical properties (for example, moduli, strength and toughness) and the elegant nano- and hierarchical structures of biological materials^1^,^2^,^3^,^4^,^5^,^6^,^7^,^11^. Various toughening mechanisms in biological materials have been proposed, including microcracking and crack bridging^3^,^12^, flaw tolerance of nanostructure^7^, viscoelastic deformation of organic layers^2^,^13^ and frictional sliding of mineral platelets^6^. In this paper, we investigate the contribution of the inherent nanoscale twins in the conch shell to its fracture toughness at the basic building block level.

Mollusk shells usually contain more than 95 wt% of hard yet brittle mineral (calcium carbonate in the form of aragonite or calcite) and only a tiny fraction of soft organics. They typically exhibit a fracture toughness several orders of magnitude higher than the corresponding single crystal of pure mineral^2^,^3^, which is widely attributed to the hierarchical structures extending from nano- to macro-scales^3^, and the staggered arrangement of mineral and organic constituents in the material^1^,^4^,^7^. The giant pink queen conch shell has a three-level hierarchical structure (Fig. 1a). The basic building block of this material is its third-order aragonite lamellae that contain a high density of nanoscale {110}-oriented growth twins aligned in parallel within a single lamella^3^,^8^,^9^,^10^. To reveal the roles of the nanoscale growth twins, *in situ* fracture experiments are conducted inside a transmission electron microscope (TEM) (Supplementary Figs 1–4) to investigate the interaction between the nanotwinned microstructure and a propagating crack in real-time, and to quantitatively measure the fracture toughness. In addition, large-scale atomistic simulations and finite element modelling (FEM) are performed to explore the underlying deformation mechanisms. Combining the results from both experiments and simulations indicate that the nanotwinned microstructure plays a key role in toughening the conch shell against crack propagation.

## Results

### Microstructural characterization

**Figure 1:**
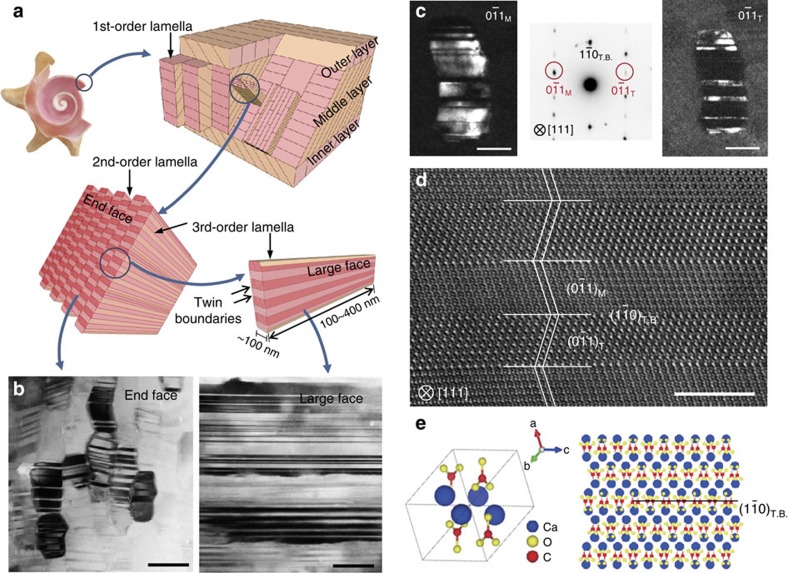
Multi-level hierarchy and nanotwinned microstructure of *Strombus gigas* conch shell. (**a**) A schematic illustration of multi-level hierarchy. Refer to Supplementary Note 1 for further detailed description. (**b**) TEM images showing the high density of nanotwins in the third-order lamellae. The nanotwins are seen from the end-face of third-order lamellae (left of panel) and the large-face (right of panel). Scale bar, 100 nm. (**c**) Dark-field TEM images of a third-order lamella taken with the 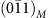 matrix (left of panel) and 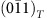 twin (right of panel) reflection, and electron diffraction pattern along [111] zone axis (middle of panel). TBs are parallel to the 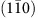 plane. Scale bar, 50 nm. (**d**) HRTEM image of nanoscale twins in a third-order lamella aligned into the [111] zone axis. Scale bar, 5 nm. (**e**) Orthorhombic unit cell of aragonite (left-hand side), and an atomic configuration of nanotwinned aragonite with 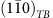 TB (right-hand side).

As illustrated in Fig. 1a, the basic building block of *Strombus gigas* is its third-order lamellae made of nanotwinned aragonite platelets 100∼400 nm long, ∼75 nm thick and ∼200 nm wide. Parallel stacking of the third-order lamellae forms a second-order lamella with width of 5–60 μm and thickness of 5–30 μm, and many second-order lamellae aggregate into a first-order lamella that is several micrometres wide and 5–60 μm thick (Supplementary Fig. 5). Figure 1b shows two TEM images of third-order lamellae seen from their large faces and end faces (Supplementary Fig. 6), which are surrounded by a proteinaceous matrix and tightly interlocked. Figure 1c shows two TEM dark-field images of a third-order lamella taken with the 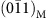
and 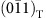 reflections, which are related by twinning on the 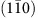 plane, and its selected area electron diffraction pattern along the [111] zone axis. Quantitative analysis of high-resolution TEM (HRTEM) images confirms that the twin thickness varies from 2 to 20 nm, with a mean value of ∼8 nm (Supplementary Fig. 7). A HRTEM in Fig. 1d indicates that the 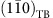 twin boundaries (TBs) are coherent and free of defects. Figure 1e illustrates the orthorhombic unit cell of aragonite and corresponding atomic configuration of a TB. The lattice rotation between twin and matrix across the 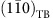 measured by geometrical phase analysis (GPA) is about 36° (Supplementary Fig. 8; Supplementary Note 2). The microstructural and crystallographic characterizations summarized in Fig. 1 reveal that the *Strombus gigas* conch shell is constructed with nanotwinned aragonite platelets as the basic building block and as such can be regarded as a nanotwinned material synthesized by nature.

### *In situ* TEM fracture testing

**Figure 2:**
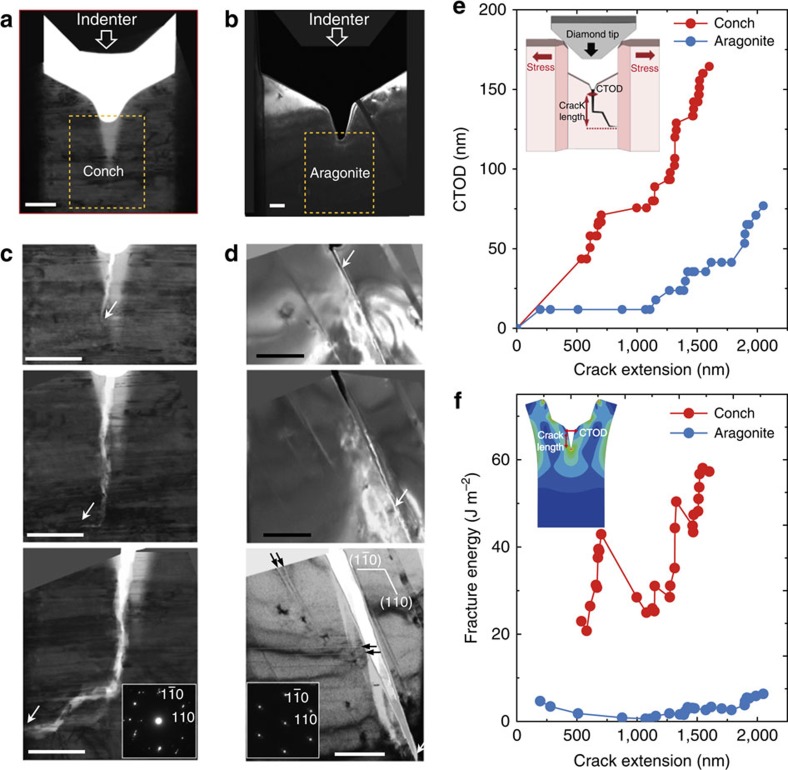
Nanoscale toughness measurement by *in situ* TEM. (**a**,**b**) TEM samples of conch shell and aragonite single crystal prepared using FIB for nanoscale toughness measurement. A flat diamond nanoindenter moves downward at a constant displacement rate (3 nm s^−1^) to initiate a crack at the notch. Details of TEM sample design and preparation are given in the Supplementary Methods. Note that dark-field TEM image is seen in **b** and bright-field TEM image is seen in **a**. Scale bar, 500 nm. (**c**,**d**) A sequence of TEM snapshots (see Supplementary Movies 1 and 2) showing the crack propagation in the conch shell and aragonite single crystal. The crack tip is marked by white arrow in each image. The black arrows indicate the deformation twins formed along the 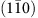 and 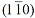 cleavage planes during the crack propagation in aragonite. The inset shows a [001] zone axis selected area electron diffraction pattern for each sample, indicating that the loading direction was parallel to the 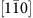 direction in both the samples. Scale bar, 500 nm. (**e**) CTOD as a function of the crack extension measured from *in situ* TEM. The inset is a conceptual representation about the measurement of CTOD and crack extension. More details are given in the Supplementary Methods. (**f**) Fracture energy versus crack extension from FEM. The inset shows a simulated sample used for FEM.

*In situ* real-time TEM fracture experiments based on a nanoindention were used to investigate crack propagation in the nanotwinned aragonite (extracted from the conch shell) and its single-crystalline (twin-free) counterpart, and to quantitatively determine the fracture toughness by measuring crack tip opening displacement (CTOD). Specially designed nanomechanical testing specimens for CTOD measurement are shown in Fig. 2a,b, where a flat diamond nanoindenter moves downward at a constant displacement rate of 3 nm s^−1^ to initiate a crack from a pre-introduced notch. Details of the TEM sample design and preparation using a focused ion beam (FIB) are given in the ‘Methods' section and Supplementary Methods. Figure 2c,d present a sequence of TEM images of crack propagation in the nanotwinned and single-crystalline aragonites, respectively. For the single-crystalline aragonite, it is observed from Fig. 2d that the main crack propagates rapidly along a cleavage plane (Supplementary Movie 2), and a few deformation twins form near the crack tip or some other stress-concentration sites (Supplementary Movie 3). However, for the nanotwinned aragonite, the main crack propagates nearly perpendicular to the TBs and is then highly deflected by the TBs, as shown in Fig. 2c (Supplementary Movie 1). More detailed TEM images (Supplementary Fig. 9) of the advancing crack tip show that the main crack can be temporarily arrested by multiple TBs, accompanied by the nucleation of a number of nanocracks ahead of its tip (Supplementary Movie 4). In contrast to the single-crystalline aragonite, the fracture path in nanotwinned aragonite is substantially rough and curved due to the blocking and deflection by the TBs.

To quantify the fracture resistance in these two specimens, we measured the CTOD based on the δ_5_ definition^14^. The details of *in situ* TEM measurement of CTOD are given in Supplementary Methods and Supplementary Fig. 4. In Fig. 2e, the measured CTOD is plotted against the crack extension distance. The crack in nanotwinned specimen exhibits a much larger CTOD than that in the single-crystalline specimen during extension. This suggests that the crack tip in nanotwinned aragonite becomes relatively blunt due to local plastic deformation, whereas the crack tip in single-crystalline aragonite remains sharp, as typical for brittle fracture. To determine the fracture toughness of the material, we also performed FEM to calculate the crack-tip J-integral (representing energy released per unit fracture surface area) following the same loading and geometrical conditions as in the experiments (see ‘Methods' section). The J-integral versus crack extension curve in Fig. 2f indicates that the fracture energy of the biogenic nanotwinned aragonite is about one order of magnitude larger than that of the twin-free aragonite single crystal. These results demonstrate that the pre-existing growth twins play an essential role in enhancing the fracture toughness of the conch shell.

Furthermore, we investigated the crack propagation path in twin-free biogenic aragonite specimen prepared from nacre, and found that the nanotwinned aragonite from conch shell has much higher fracture resistance (Supplementary Note 3, Supplementary Fig. 9 and Supplementary Movie 5). This is particularly interesting because nacre contains 5 wt% protein while the conch shell contains only 1 wt% protein. Previous experimental studies on the fracture of mollusk shells have shown that the soft organic phase plays a dominant role in preventing crack propagation^2^,^15^. However, the conch shell, known as the most highly mineralized mollusk shell, has less volume/weight fraction of organics but higher fracture toughness than nacre. This is consistent with our *in situ* TEM observations and analysis in suggesting that the nanoscale growth twins are critical in allowing the conch shell to achieve an extraordinarily high fracture toughness with minimal organic content.

### Nanotwin-governed toughening mechanisms

**Figure 3:**
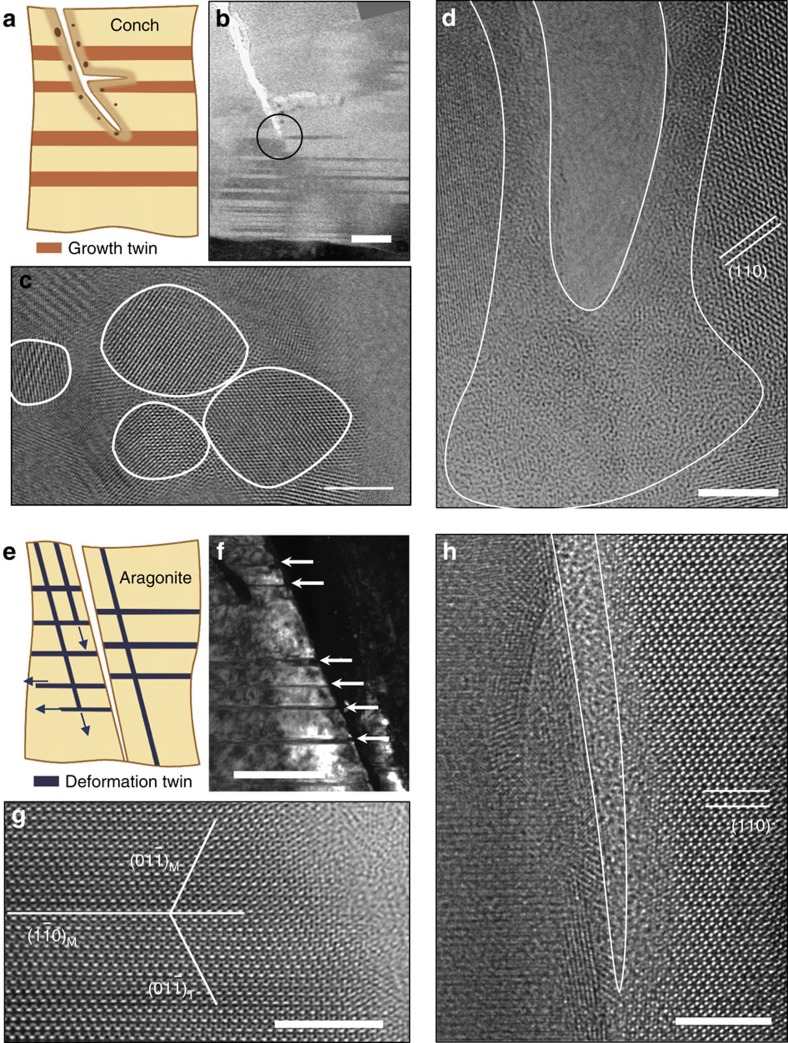
Nanotwin-governed toughening mechanisms observed in experiments. (**a**) Schematic illustration of toughening mechanisms in the nanotwinned aragonite of conch shell, including crack-tip branching, blunting and phase transformation. The formation of amorphous and nanograins are highlighted as different colour along the periphery of the crack. (**b**) TEM image showing a crack tip arrested by nanoscale growth twins in the conch shell. Scale bar, 50 nm. (**c**,**d**) HRTEM images of the edge of fractured surface of the conch shell. Some of the nanograin and amorphous phases are outlined by white lines in **c** and **d**, respectively. Scale bar, 5 nm. (**e**) Schematic illustration of crack tip plasticity (deformation twinning) in single-crystalline aragonite. The deformation twins are formed along the 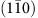 and 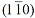 planes. (**f**) TEM image of a typical crack in single crystal aragonite. Some deformation twins nucleated from crack tip are indicated by white arrows. Scale bar, 50 nm. (**g**) HRTEM image of a deformation twin formed along the 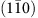
plane. Scale bar, 5 nm. (**h**) HRTEM image of crack tip in single-crystalline aragonite. No phase transformation is observed along the atomically cleaved fracture surfaces. Scale bar, 5 nm.

**Figure 4:**
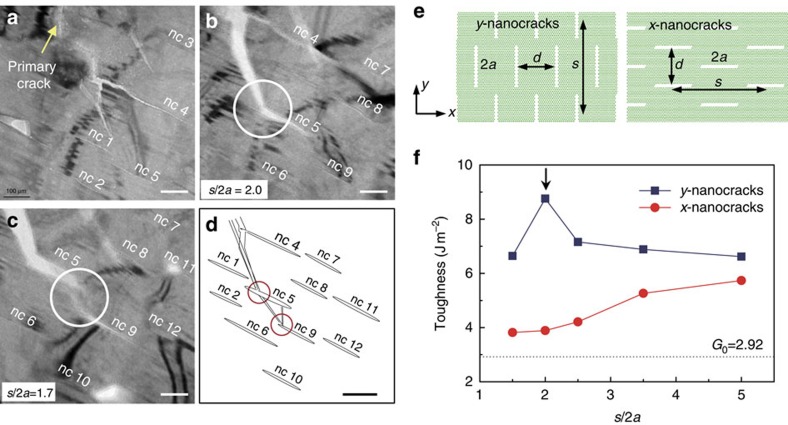
Nanocrack toughening. (**a**) Initiation of a primary crack inclined to TBs on large faces and formation of multiple nanocracks (indicated as ‘nc' followed by number) which span over hundreds of nanometres in the vicinity of the primary crack. Scale bar, 100 nm. (**b**,**c**) TEM image showing the propagation of the primary crack through multiple nanocracking (for example, nc 5 and nc 9), resulting in an increased resistance against catastrophic propagation (Supplementary Movie 4). Scale bar, 100 nm. (**d**) Schematic illustration of the nanocrack pattern in **a**–**c**. Scale bar, 100 nm. (**e**) Simplified model patterns of nanocracks used in the coarse-grained simulations: one perpendicular (referred to as *y*-nanocracks) and the other parallel (*x*-nanocracks) to the primary crack. The parameters used to describe the nanocrack pattern are: *d* for crack spacing, 2*a* crack length and *s* crack period. (**f**) Variation of toughness with a dimensionless parameter *s*/2*a*.

To identify the underlying deformation mechanisms, the microstructural changes near the crack tip were investigated carefully by using HRTEM after the *in situ* TEM fracture experiments. In addition, dedicated *in situ* TEM nanoindentation experiments were carried out to observe the deformation mechanisms in real-time (Supplementary Fig. 2, and Supplementary Movies 6 and 7). Figure 3b shows a representative TEM image of an advancing crack tip in the nanotwinned aragonite, which is temporarily arrested by a pre-existing TB, indicating that TBs can effectively block the crack propagation. Meanwhile, a nanocrack is nucleated and links up with the main crack, leading to crack branching, as illustrated in Fig. 3a. The influence of nanocracks on fracture toughness is discussed in detail in the context of Fig. 4. The HRTEM images in Fig. 3c,d further reveal that there exists a mixture of amorphous and nanocrystalline phases around the crack tip. Such amorphization and formation/re-orientation of nanograins are induced by the elevated stress field in the vicinity of the crack tip (Supplementary Movie 7). It indicates that when a crack is trapped by a TB, the region around the crack tip can undergo severe plastic deformation. This is further demonstrated by apparent crack blunting in Fig. 3d. For the single-crystalline aragonite, deformation twinning is found to be operative near the crack, as revealed by Figs 2d and 3e,f (Supplementary Movies 2 and 6). Some deformation twins are captured in Fig. 3f, and the corresponding twin-matrix crystallographic relation is shown in the HRTEM image (Fig. 3g). The TEM diffraction patterns show that {110}-type deformation twinning is a dominant mode in single-crystalline aragonite (Supplementary Figs 10–11). Although deformation twinning can dissipate energy to some extent, it cannot effectively hinder crack propagation, as evidenced by the sharp crack tip in Fig. 3h and the catastrophic cleavage fracture shown in Fig. 2d (also see Supplementary Movies 2 and 3). A HRTEM examination shows that no phase transformation occurs near the crack tip in the single-crystalline aragonite (Fig. 3h). The differences in crack-tip deformation modes between nanotwinned and single-crystalline aragonite indicate that the pre-existing TBs of growth twins in nanotwinned biogenic aragonite are uniquely effective in blocking crack advance and enhancing energy dissipation via phase transformations in the vicinity of the crack tip, leading to higher fracture toughness shown in Fig. 2e,f.

### Nanocrack toughening

More detailed TEM observations reveal that a few nanocracks are formed in the vicinity of the primary crack tip with a distinct pattern (Fig. 4a–d; Supplementary Movie 4). Here we also evaluated the contributions of multiple nanocracking to the toughness using coarse-grained simulations based on a triangular lattice model (see Supplementary Methods). In the coarse-grained simulations, the crack period *s* varies from 300 to 1,000 nm, whereas the crack length 2*a* and spacing *d* remain constant at the observed values (2*a*=200 nm and *d*=200 nm). Two simplified model patterns of nanocracks were considered, one perpendicular (referred to as *y*-nanocracks) and the other parallel (*x*-nanocracks) to the primary crack (Fig. 4e). The simulations show that the nanocracks, while reducing the elastic modulus, enhance the toughness of the material by a factor from 1.3 to 3 by shielding the main crack compared with the homogenous material (Fig. 4f), which is consistent with previous theoretical predictions^16^,^17^,^18^,^19^. More interestingly, an optimal crack pattern (indicated by an arrow in Fig. 4f) yielding the maximum toughness from the current simulations for *y*-nanocracks (*s*/2*a*=2.0) is very close to that observed in the current *in situ* TEM experiments (Fig. 4a–d; Supplementary Movie 4). More details of the simulations and the relevant results are given in Supplementary Methods, Supplementary Note 4 and Supplementary Figs 12–14. A combination of the above experimental measurement and the results from the coarse-grained simulations suggests that it is the crack-blocking effect of TBs, rather than multiple nanocracking, that plays the dominant role in the toughening of conch shell.

### Atomistic simulations of crack propagation

**Figure 5:**
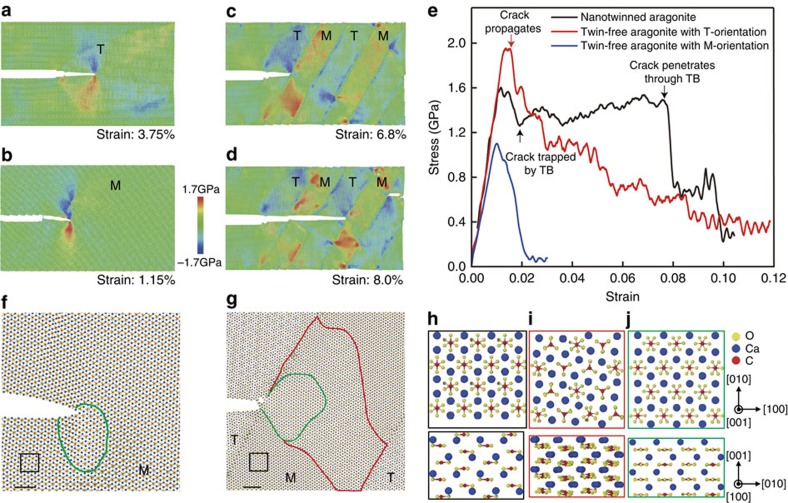
Atomistic simulations of crack propagation in nanotwinned and twin-free aragonites. (**a**,**b**) Snapshots of crack propagation in twin-free aragonite single crystals with different orientations under strains of 3.75% and 1.15%, respectively. The two orientations, denoted by ‘M' and ‘T', are related by 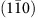 twin relationship. (**c**,**d**) A sequence of snapshots of crack propagation in a nanotwinned aragonite under strains of 6.8% and 8.0%, respectively. (**e**) Stress–strain curves of nanotwinned and twin-free aragonites with the edge crack. (**f**,**g**) Structural transformation at the crack tip in single-crystalline and twinned aragonite. The perfect matrix regions are outlined by black lines, while the transformed regions are outlined by green and/or red lines. Scale bar, 2 nm. (**h**–**j**) Typical atomic configurations of aragonite near crack tip before and after structural transformation. The pictures in **h**–**j** illustrate the atomic structures of the regions outlined by black, red and green lines in **f** and **g**, respectively. The atomic structures in upper panel are seen from the [001] direction while those in lower panel are seen from the [100] direction.

To gain further insights into the atomistic mechanisms of how the nanotwinned microstructure enhances fracture resistance, we have performed large-scale molecular dynamics (MD) simulations of crack propagation in both nanotwinned and twin-free aragonites. The nanotwinned sample used in our simulations has the TB spacing of 10 nm as in the conch shell (Supplementary Movie 8). The sample with TB spacing of 20 nm was also tested to check the twin size effect on fracture toughness (see Supplementary Note 5, Supplementary Fig. 15 and Supplementary Movie 9). In these nanotwinned samples, a crack is created on the (010) plane along the [100] direction, a cleavage direction of aragonite, which is inclined with respect to the 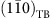. For the twin-free single-crystalline aragonite, we constructed two typical samples: one has the same crystallographic orientation as the twin domain (Fig. 5a and Supplementary Movie 10), and the other has the orientation of the matrix (Fig. 5b and Supplementary Movie 11), which are conveniently referred to as samples with T- and M-orientations, respectively. More details of the simulations are provided in the ‘Methods' section.

In the twin-free aragonite with T- and M-orientations, deformation is localized in the vicinity of the crack tip, and the crack propagates smoothly, as evidenced by Fig. 5a,b. In contrast, the nanotwinned aragonite exhibits a very distinct fracture behaviour; the crack is always trapped at TBs for some periods of time and the crack tip becomes blunted, due to sliding along the TB (Fig. 5c,d and g), which are very similar to the experimental observations (Figs 2c and 3b–d and Supplementary Fig. 9a–f). The in-plane stress contours in Fig. 5c,d further reveal substantial stress/strain delocalization (alternating stresses with opposite signs) around the crack tip. These phenomena are also revealed by the FEM simulations (see Supplementary Methods and Supplementary Fig. 16). The results from both atomistic and FEM simulations demonstrate that TBs can act as effective obstacles to crack propagation, leading to crack blunting and delocalization of deformation, which facilitates energy dissipation and delays catastrophic crack propagation.

Our atomistic simulations not only showed phase transformation near the crack tip (Fig. 5f,g), but also revealed the detailed mechanism involving a coordinated rotation of some carbonate groups (CO_3_) over the transformed region driven by the crack-tip stress field, as can be seen from the typical atomic configurations of aragonite before and after the transformation (Fig. 5h–j). Such phase transformation is irreversible, that is, the transformed regions cannot spontaneously recover upon unloading. Similar stress-driven phase transformation was also observed at the crack tip of nanotwinned aragonite under the nanoindenter (Fig. 3c,d) and in previous MD simulations of calcite deformation under high pressure^20^. It is noted that in the single-crystalline aragonite, the phase transformation occurs before crack initiation, and the transformed region is highly limited (Fig. 5f, Supplementary Note 6, Supplementary Figs 18,19 and Supplementary Table 1). In contrast, for the nanotwinned aragonite, large-scale phase transformation is activated once the crack is trapped by a TB and spreads through the nanoscale twins (Fig. 5g), resulting in delocalization of plastic deformation.

Figure 5e shows the stress–strain curves from our present simulations of nanotwinned and twin-free aragonites. The plateaus in the stress–strain curve of nanotwinned aragonite correspond to the crack being trapped by TBs. On the basis of the calculations in Supplementary Methods, the fracture energies of twin-free aragonites with T- and M-orientations are 0.55 and 0.28 J m^−2^, respectively. Remarkably, the fracture energy of the nanotwinned aragonite with alternating T- and M-orientations is found to be around 5.13 J m^−2^, which is approximately an order of magnitude higher than that of the twin-free aragonite. This dramatic enhancement in fracture toughness of nanotwinned aragonite is consistent with our experimental measurements, and the simulation results provide a strong evidence that the toughening effect originates from TBs impeding crack propagation and inducing delocalized plastic deformation. In addition, we have also conducted MD simulations of crack propagation in nanotwinned samples with TBs parallel to the initial crack (Supplementary Note 7 and Supplementary Fig. 20). The results indicate that the presence of TBs also significantly increases the fracture toughness of aragonite even if the TBs are parallel to the initial crack. This suggests that the relative orientation between the TBs and crack is a factor that affects the fracture resistance, and points to the importance of crossed lamellar stacking in upper level hierarchy (that is, first- and second-order lamellae) in hindering crack propagation from different directions.

### Phase transformation in nanotwinned aragonite

**Figure 6:**
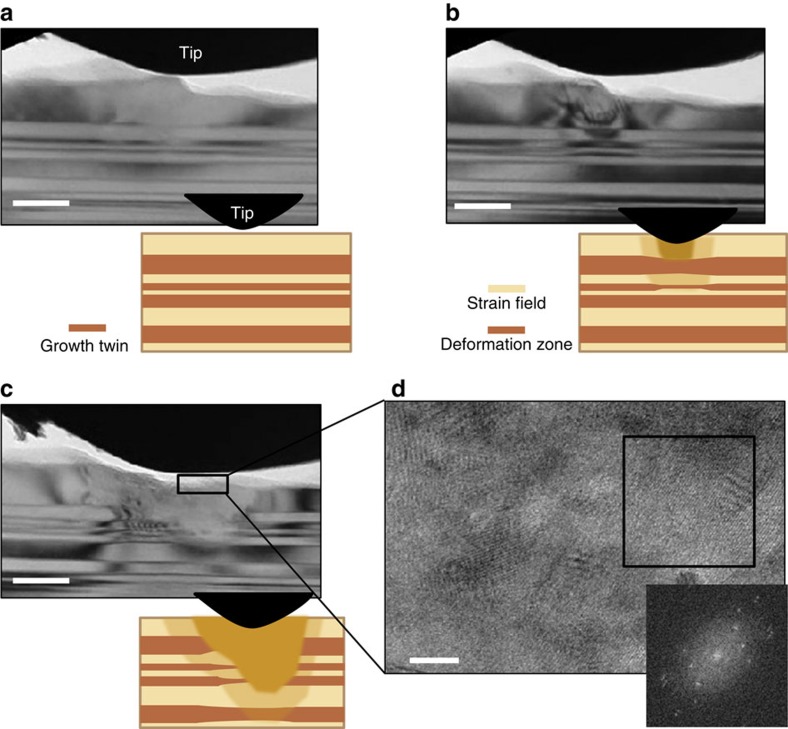
Stress delocalization and energy dissipation by phase transformation in nanotwinned aragonite under nanoindentation. (**a**) TEM image and schematic illustration of nanoscale growth twins before nanoindentation in TEM. Scale bar, 50 nm. (**b**,**c**) TEM images and schematic illustrations showing the evolution of the deformation zone during nanoindentation (see Supplementary Movie 7). The strain field and the deformation zone consisted of amorphous phase and nanograins are highlighted in different colours. During the stress-induced local phase transformation, the nanotwins close to the transformed region show a slight change in the TB spacing. Scale bar, 50 nm. (**d**) HRTEM image of the deformation zone showing the formation of nanograins through phase transformation. A fast-Fourier transformed pattern obtained from the boxed region is shown as inset in **d.** Scale bar, 10 nm.

For man-made nanotwinned metals, the migration of pre-existing TBs due to nucleation and slip of partial dislocations on TBs is a softening mechanism that significantly contributes the ductility of material^21^,^22^,^23^. However, for the nanotwinned aragonite from conch shells, our current *in situ* TEM observations (Supplementary Movie 7) showed that the pre-existing 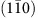 TBs do not migrate during nanoindentation. Figure 6a–c showed a sequence of TEM images of the structural evolution of nanotwinned aragonite during nanoindentation, indicating that some nanoscale twins under an indenter are gradually destroyed, but TBs do not pronouncedly migrate during loading. HRTEM image and associated FFT pattern in Fig. 6d revealed that the damage of nanoscale twins is associated with the structural transformation in severe local deformation zone. This can be understood as a consequence of the change of atomic arrangement due to the rotation of carbonate groups under the local high stress level, as illustrated in Fig. 5h–j. In the current study, inactivation of the migration of pre-existing TBs might be due to two factors. First, TBs are nearly perpendicular to the loading direction, so that there is not enough resolved shear stress to drive TB migration. Second, the strong ionic bonding structure might give rise to good stability of nanoscale twins and high critical stress for dislocation nucleation on pre-existing TBs.

## Discussion

Previous theoretical models^24^,^25^,^26^ have predicted that the fracture toughness of self-similar hierarchical materials increases exponentially with the number of hierarchical levels, and this has also been demonstrated by experimental and computational studies^27^,^28^. To further validate/complement the results from both experiments and simulations, we have extended a theoretical model of the self-similar hierarchical materials^26^. The relevant details of theoretical model are supplied in Supplementary Note 8. According to a bottom-up design route of the self-similar model^26^, an optimal three-level hierarchical structure, referred to as a conch-like material, is built by determining the geometry and mechanical properties (including the stiffness, strength and toughness) of all the levels. We evaluated the contribution of nanoscale twins in the third-order lamellae to the overall fracture toughness of the conch-like hierarchical structure. As shown in Supplementary Fig. 17c, the overall toughness of the conch-like hierarchical material increases dramatically as the lowest-level mineral fracture energy is enhanced. Supplementary Fig. 17d provides a direct prediction that if the fracture toughness and strength of the lowest-level structure are increased only two to three times, the overall toughness of the hierarchical material will increase by one order of magnitude. These results indicate that the lowest-level structure plays a crucial role in increasing the overall toughness of the conch-like hierarchical material by driving energy dissipation in the organic matrix at all the upper levels.

Our experiments and simulations have shown that nanoscale twins enhance the fracture energy of the lowest-level structure in the aragonitic shell of *Strombus gigas* by about one order of magnitude. In comparison, the overall fracture energy of the conch shell is known to be about three orders of magnitude higher than that of the single-crystalline aragonite. Together with the above theoretical analysis, we conclude that the nanotwin-toughened-third-order-lamellae are able to drive larger scale energy dissipation in the second- and first-order lamellae structures, leading to an exponential increase in the overall fracture energy of the material as we move up in hierarchy, in agreement with the existing theoretical models on self-similar hierarchical materials.

Previous experimental studies^29^,^30^,^31^ have shown that deformation twinning is a controlling mechanism during plastic deformation of geological aragonite and calcite crystals. In our current study, deformation twinning is observed to be a dominant energy-dissipation mechanism during crack propagation of twin-free single-crystalline aragonite, as evidenced by Figs 2d and 3e–h. In contrast, in the case of biogenic nanotwinned aragonite in the conch shell, our study based on both experiments and atomistic simulations has shown that no additional twinning was activated during the fracture. Rather, it is TBs' blocking crack and phase transformation (including amorphization and formation/reorientation of nanograins) that act as the controlling mechanism of toughening. The inactivation of deformation twinning in biogenic nanotwinned aragonite is attributed to the presence of nanoscale pre-existing twins, which apparently have suppressed the formation of additional twins near a crack tip.

Notably, there are many different species of mollusk shells in nature that exhibit different toughening mechanisms. Some of the bivalve shells, such as the brachiopod shells and *Placuna placenta* shells, possess more than 90 wt% calcitic mineral which, like nacre, is free of twins. A very recent study on nanoindentation of *Placuna placenta* shell^32^ has shown that profuse deformation twinning occurs in the biogenic calcite, leading to an enhancement of energy dissipation by about an order of magnitude, compared with the single-crystalline calcite mineral. It was found that while deformation twinning is spatially localized under nanoindentation, it catalyses a variety of additional inelastic mechanisms^32^,^33^, which effectively dissipates a considerable amount of energy under predatory attacks and makes the calcite-based shells resistant to catastrophic fracture. In comparison, in the aragonite-based conch shell, the crack-blocking effect of TBs and the resulting delocalization of deformation by phase transformation are identified as the dominant toughening mechanisms. It is also interesting to note that the twins in *Strombus gigas* shell are inherent, about 2–20 nm thick and distribute uniformly within the basic building blocks of aragonite lamellae, while the twins in *Placuna placenta* shell are produced by deformation, ∼50 nm thick and localize only in the deformed or damaged zone. Therefore, in the aragonite- and calcite-based shells, the nanoscale twins (from growth or deformation twinning) play a common and crucial role in maintaining sufficient toughness to prevent fracture, but the difference in microscopic deformation mechanisms essentially reflects that nature has evolved different energy-dissipating strategies to synthesize bio-composites with unique mechanical properties.

During the past decade, it has been demonstrated that nanoscale twins embedded in micron- or sub-micron-sized grains of polycrystalline metals induce an exceptional combination of mechanical and physical properties including ultra-high strength and hardness, good tensile ductility and strain hardening, superior fatigue and wear resistance^21^,^34^,^35^,^36^,^37^,^38^. Recently, nanotwinned cubic boron nitride and diamond have been synthesized in the laboratory^39^,^40^, with unprecedented hardness and toughness. Pre-existing growth twins play a key role in the mechanical properties of these man-made nanotwinned materials. For instance, in nanotwinned metals, both high strength and ductility can be attained as the TBs effectively impede dislocation motion but still allow a high density of mobile dislocations to operate in a highly organized lamellar structure^21^,^34^,^35^,^36^,^37^,^38^. Our present work has demonstrated the crucial role of biogenic growth twins in the mechanical properties of biological materials. Although the nanotwinned structure seems beneficial for both engineering and biological systems, the toughening mechanisms in biogenic nanotiwnned aragonite are distinct from those observed in man-made nanotwinned metals. For example, a recent *in situ* TEM study^38^ revealed that, in nanotwinned metal foils, dislocations are emitted from a moving crack-tip and impinge on TBs ahead of the crack, leading to the formation of dislocation walls on the TBs which become impenetrable to further dislocation motion and hinder the propagation of the crack. In contrast, the mineral in biological materials has ionic bonding and is nominally brittle, and the underlying mechanisms for energy dissipation have been identified here as crack trapping at TBs, multiple nanocracking, phase transformation and strain delocalization driven by the crack-tip stress field.

In summary, the present study clearly indicates that nanoscale growth twins in *Strombus gigas* conch shell play a critical role in the mechanical properties of the material. *In situ* nanoindentation-based fracture experiments on both nanotwinned and twin-free specimens from *Strombus gigas*, nacre and single-crystalline aragonite reveal that the TBs of nanoscale growth twins can effectively block crack propagation and induce phase transformation in the vicinity of the crack-tip, leading to the delocalization of deformation and efficient energy dissipation that delay catastrophic fracture, which is corroborated by atomistic simulations and FEM modelling. The results from both experiments and simulations show that the nanotwinned microstructure improves the fracture toughness of aragonite by roughly an order of magnitude. These findings provide a fundamental understanding of the contribution of hard basic building blocks at nanoscale to fracture resistance of the overall structure, which complement our previous knowledge about the roles of soft organic content and bio-composite structure in the toughness of bio-materials. The present study may also have profound impact on the design of strong and tough biomimetic materials with minimum organic content.

## Methods

### Transmission electron microscopy

Dry and externally cleaned *Strombus gigas* conch shells were cut normal to the growth line (shell axis) to expose three macroscopic layers in the cross-section of the outer whorl. The sectioned sample was sliced and polished down to 30 μm (MultiPrep System 15-2000, Allied) at 45° with respect to the shell surface to expose the end face and the large face of third-order lamellae in an edge-on projection (Supplementary Fig. 1a). The sample was then thinned to less than 1 μm by mechanical wedge polishing (Model 590 Tripod Polisher, South Bay) (Supplementary Fig. 1b,c). The wedge polished sample was attached to a half-cut 3 mm Cu grid using epoxy (see, for example, Supplementary Fig. 2d). Using an Ar^+^ ion miller (PIPS 691, Gatan, Inc.), it was further thinned for electron transparency at liquid nitrogen (LN_2_) temperature. To remove any surface damage layer that might be produced by the ion milling, low energy Ar^+^ ion milling was carried out at 250 V for 10 min using Gentle Mill (Technoorg Linda). Finally, thin layers of carbon were coated on both sides of the TEM sample to make it conductive to the electron beam. The TEM specimens of nacre and single-crystalline aragonite were prepared following the same method. A field-emission TEM (JEM-2100F, JEOL) operated at 200 kV and a high voltage TEM (JEM-1300S, JEOL) operated at 1.25 MV were used for the conventional microstructure analysis and the atomic structure of nanoscale twins and the fractured surfaces, respectively.

### *In situ* fracture testing in TEM

Notched TEM samples for CTOD measurement were prepared using a focused ion beam (FIB, Helios Nano-Lab, FEI). To precisely control the loading axis for *in situ* TEM nanomechanical testing, the samples cut from *Strombus gigas* conch shell and single crystal aragonite were polished to expose a specific crystallographic orientation before FIB processes. In the case of *Strombus gigas* conch shell, the polishing was conducted along the large face of the third-order lamellae to expose the 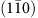 TBs parallel to the surface. The 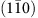-orientated surface of aragonite single crystal was prepared for comparison. A thin lamella was milled using a high energy Ga^+^ ion beam in FIB and lifted out from the polished surface and attached to a Cu grid through Pt deposition. Subsequently, the sample design shown in Supplementary Fig. 3b was patterned in top-view using a beam current of 93 pA at 30 kV. Fine machining was carried out in side view using a beam current of 48 pA at 30 kV (Supplementary Fig. 3f). As a final step, low energy milling was performed at beam voltages of 5 and 1 kV to remove surface damages and/or contamination caused by the high energy Ga^+^ ion beam. The details on sample preparation are summarized in Supplementary Fig. 3. *In situ* fracture tests with CTOD measurement were carried out in a TEM (JEM-2100F, JEOL) operated at 200 kV by using a single tilt nanoindentation holder (Nanofactory). The holder is equipped with a flat-ended diamond indenter and a piezo-stage to which a TEM specimen is mounted for precise positioning and quantitative manipulation of displacement. The diamond indenter was machined using FIB to fit into the notch of the TEM sample. The sample was mounted to the piezo-stage with great care to reduce the misalignment between the tip and the sample. All the mechanical tests were performed in displacement control mode at a displacement rate of 3 nm s^−1^. Dynamic events of crack initiation and propagation were recorded in real-time by using a charge-coupled device camera (ORIUS 200D, Gatan) at 25 frames per second. For *in situ* nanoindentation tests in TEM, the wedge-polished TEM samples and a sharp diamond indenter were used. The TEM specimen was bonded to a Au wire using silver epoxy and assembled into the piezo-stage of the nanoindentation holder. After making contact between the sample edge and the indenter tip, the indentation test was conducted at a typical displacement rate of 10 nm s^−1^.

### FEM calculation of J-integral according to CTOD

Two-dimensional samples (Supplementary Fig. 21) were constructed in ABAQUS following the same loading and geometrical condition as in the *in situ* TEM experiments. Aragonite was modelled as a homogeneous elastic material with Young's modulus of 120 GPa and Poisson's ration of 0.3. The pre-existing crack was set to different lengths according the CTOD measurements. The J-integral value was calculated after loading until the CTOD in FEM matched with the experiment data.

### Atomistic simulations

Three aragonite samples with controlled twin orientations were used for the simulations: a twin-free single-crystalline aragonite in *T*-orientation, a twin-free aragonite in *M*-orientation and a nanotwinned aragonite with alternating *T*- and *M*-orientations (referred to as nanotwinned aragonite) in which TBs are slanted with respect to an advancing crack along a cleavage plane. Each sample is about 50 nm wide, 100 nm long and 1.1 nm thick and contains about 0.5 million atoms. All the MD simulations were performed using software package LAMMPS^41^. Interatomic forces in aragonite are described by an empirical potential^42^, which includes Coulomb, LJ, harmonic angular and dihedral interactions. All the simulated samples were initially relaxed and equilibrated at 5 K for 50 ps using Nosé–Hoover thermostat and barostat^43^. Periodic boundary condition is imposed along the thickness direction of the samples. After relaxation, the mode I loading is applied at a constant strain rate of ∼2 × 10^8^ s^−1^. During loading, an NVT ensemble with Nosé–Hoover thermostat^43^ is used to maintain a constant temperature. Visualization is processed via software package Ovito^44^.

## Additional information

**How to cite this article:** Shin, Y. A. *et al*. Nanotwin-governed toughening mechanism in hierarchically structured biological materials. *Nat. Commun.* 7:10772 doi: 10.1038/ncomms10772 (2016).

## Supplementary Material

Supplementary Information Supplementary Figures 1-21, Supplementary Table 1, Supplementary Notes 1-8, Supplementary Methods and Supplementary References

Supplementary Movie 1*In-situ* TEM nanoscale CTOD measurements of conch shell. The results of two separate tests are displayed in series. The TEM movies were recorded at 25 fps and played at 37.5 fps (1.5 times faster playback speed) for the first movie (Conch A, analyzed and presented as part of Fig. 2) and at 25 fps for the second movie (Conch B). The mechanical testing was conducted at a constant displacement (3 nm/s) of the FIB-machined diamond indenter normal to the 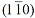 TBs. After being initiated at the notch, the crack exhibits several branching and deflection as it propagates across the nanoscale twins. The plot of CTOD versus crack extension is displayed in sync with the corresponding movie. After playback of each TEM movie, the TEM images taken after the test are shown to highlight the crack propagation path.

Supplementary Movie 2*In-situ* TEM nanoscale CTOD measurements of single crystal aragonite. The results of two separate tests are displayed in series. The TEM movies were recorded at 25 fps and played at the same playback speed for the first movie (Aragonite A), and at 37.5 fps (1.5 faster playback speed) for the second movie (aragonite B, analyzed and presented as part of Fig. 2). The mechanical testing was conducted at a constant displacement (3 nm/s) of the FIB-machined diamond indenter along the 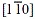 direction. Once being initiated, the crack propagates all the way along the 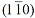 cleavage plane without much hindrance. During the crack propagation, deformation twins occur along the as well as the 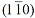 planes near the crack tip. The plot of CTOD versus crack extension is displayed in sync with the corresponding movie. After playback of each TEM movie, the TEM images taken after the test are shown to highlight the crack propagation path and also the formation of deformation twins.

Supplementary Movie 3Deformation twinning during crack propagation in single crystal aragonite. Deformation twinning occurs ahead of crack tip as a major plastic deformation induced by the crack tip stress fields. The deformation twins form and propagate along the 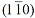
and planes with width of a few nm.

Supplementary Movie 4 Formation of nanocracks and their interaction with main crack in the conch shell. The advancing main crack was blocked frequently by the slanted TBs. While the main crack is trapped at the TBs for a while, nanocracks (marked by yellow arrows in the movie) were nucleated ahead of the main crack. The main crack propagated through repeated deflection and coalescence with the nanocracks. This observation inspired coarse-grained simulations to reveal the optimum spacing and size of nanocracks (Supplementary Method 5). The observed crack pattern is very close to one that yields the highest fracture toughness in the simulation (Supplementary Discussion 7).

Supplementary Movie 5Crack propagation paths in conch shell, nacre, and single crystal aragonite. The plot of crack length versus loading time is displayed in sync with the corresponding TEM movie. In the conch shell, the main crack propagated through crack deflection and coalescence with nanocracks (indicated by yellow allows). In the case of nacre, a single crack propagated through the aragonite plates without hindrance (shown as steep ramps of the curve), while being trapped or deflected at the organic interfaces between adjacent plates (plateau regions in the curve). In contrast, crack propagated in single crystal aragonite in a brittle manner along the 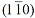 cleavage planes. The movie tracks the fracture surface after the crack propagated all the way through.

Supplementary Movie 6Deformation twinning in aragonite during *In-situ* TEM nanoindentation. Deformation twinning occurred under the indenter as a major plastic deformation driven by the stress fields. The loading axis was parallel to the 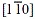 direction of aragonite. The deformation twins formed and propagated along the 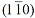 and (110) planes with width of a few nm. In addition to deformation twinning, structural transformation (or amorphization) was also seen to occur under the indenter.

Supplementary Movie 7Phase transformation in conch shell during *In-situ* TEM nanoindentation. The stress fields under the indenter induced a type of phase transformation (or amorphization) of nanotwinned aragonite with no indication of dislocation slip or deformation twinning. The loading axis was normal to the 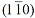 TBs of nanotwinned aragonite sampled from the conch shell. Unlike a single crystal aragonite, no crack was formed within the same amount of displacement in the nanoindentation test.

Supplementary Movie 8 MD simulation of crack propagation in nanotwinned aragonite (with twin boundary spacing of 10nm). Under increasing loading, an edge crack starts to propagate toward an array of TBs. When the crack reaches a TB, it is trapped by the TB over a time period. The crack tip becomes blunted due to sliding on the TB. Plastic deformation via structural transformation ahead of the crack tip is confined within the nanoscale twins. As the applied loading continues, the crack eventually penetrates through the TB.

Supplementary Movie 9 MD simulation of crack propagation in nanotwinned aragonite (with twin boundary spacing of 20nm). The results are qualitatively similar to those in Supplementary Movie 8.

Supplementary Movie 10 MD simulation of crack propagation in single-crystalline aragonite in T-orientation. Structural transformation occurs ahead of the crack tip while the crack propagates in a straight path.

Supplementary Movie 11MD simulation of crack propagation in single-crystalline aragonite in M-orientation. The crack is slightly deflected along a 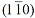 cleavage plane during the propagation and the transformed region driven by the crack-tip stress field is smaller than that in single-crystalline aragonite samples in T-orientation.
