# Supplementary material for: Nanotwin-governed toughening mechanism in hierarchically structured biological materials
Source: Nat Commun. 2016 Feb 17;7:10772. doi: 10.1038/ncomms10772 (PMC4757792; doi:10.1038/ncomms10772)
Supplement: Supplementary Information — Supplementary Figures 1-21, Supplementary Table 1, Supplementary Notes 1-8, Supplementary Methods and Supplementary References [file ncomms10772-s12.pdf]

Supplementary information

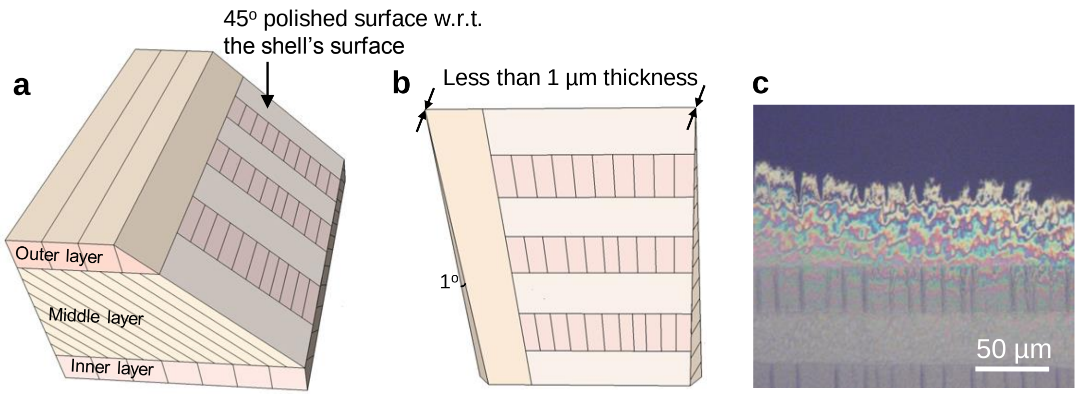

Supplementary Figure 1 | TEM specimen preparation for conventional TEM and high-resolution TEM (**a**) Schematic illustration showing the polishing orientation. (**b**) Schematic illustration of a wedge polished specimen. The wedge angle is about 1º and the thickness of the sample edge is less than 1 μm. (**c**) Optical microscopy image showing a prepared TEM sample after Ar^+ ion milling.

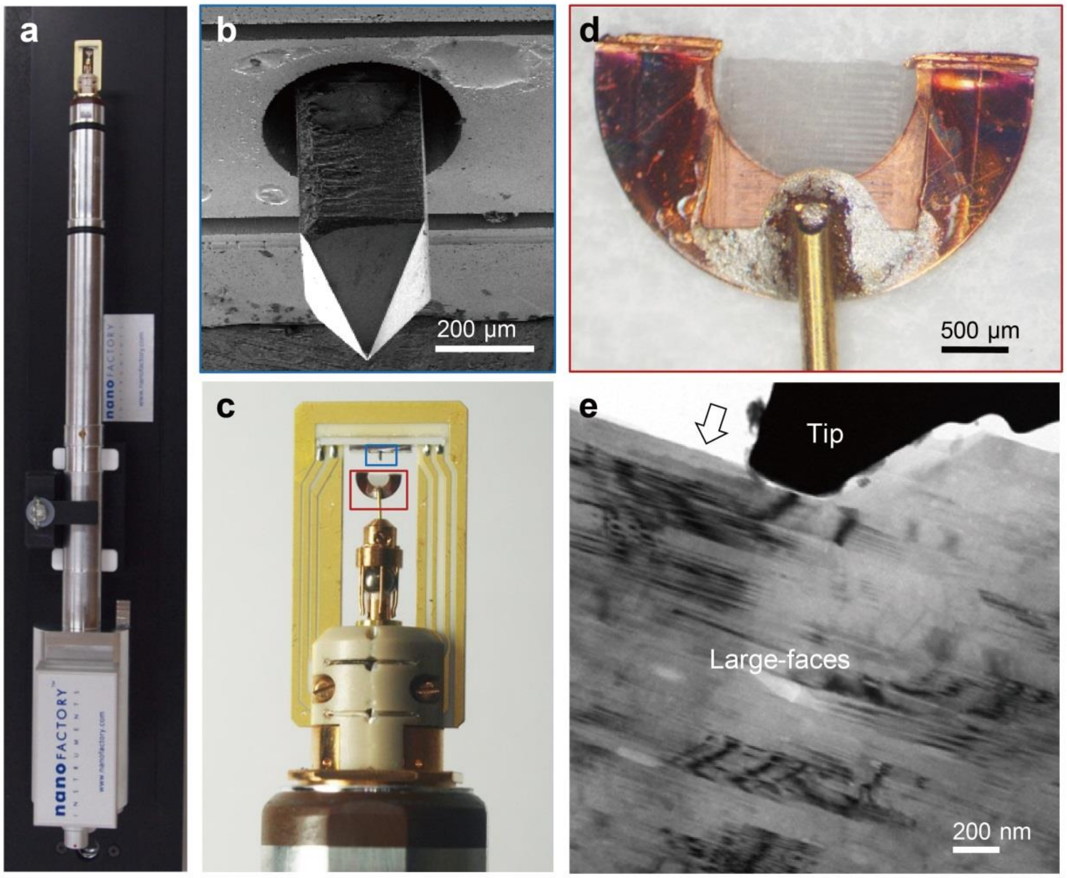

Supplementary Figure 2 | In-situ TEM Nanoindentation holder and a wedge polished TEM sample for *in situ* TEM nanoindentation. (**a**) A single-tilt nanoindentation TEM holder (Nanofactory^{TM}). (**b**) SEM micrograph of a diamond indenter installed in the TEM holder. (**c**) Magnified view of the diamond indenter (blue-lined box) and TEM sample (red-lined box) mounted on the piezo-stage. (**d**) TEM specimen prepared by following the mechanical wedge polishing method described in Supplementary Methods. (**e**) Loading geometry of the diamond indenter to the sample edge. Loading direction is indicated by a white arrow.

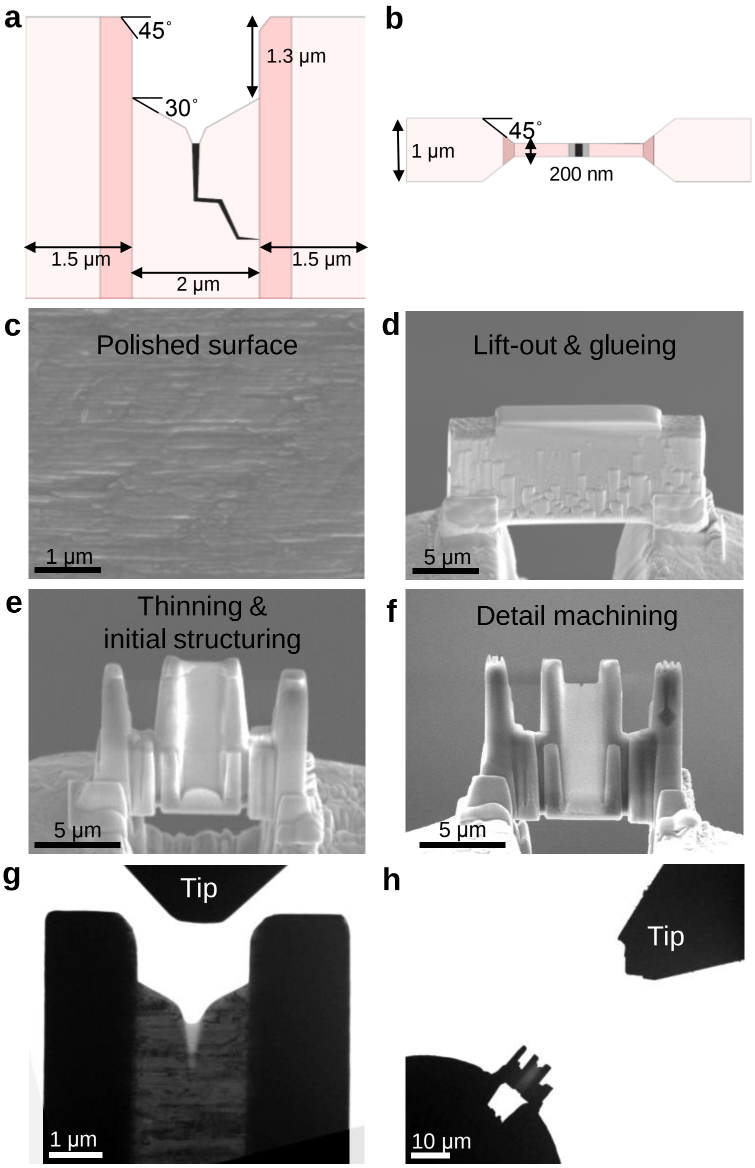

Supplementary Figure 3 | Design and fabrication of *in situ* TEM Specimen for nanoscale CTOD measurement. Schematic illustrations of TEM specimen design for CTOD measurement in (**a**) side view and (**b**) top view. (**c**) SEM micrograph of a mechanically polished surface of conch shell. (**d**) Fixing of the lift-out sample on a Cu support grid. (**e**) Initial structuring. (**f**) Detailed machining. (**g**) Experimental setup for CTOD measurement in TEM with a flat-ended nanoindentation tip. (**h**) TEM image showing the alignment between a diamond tip and the specimen.

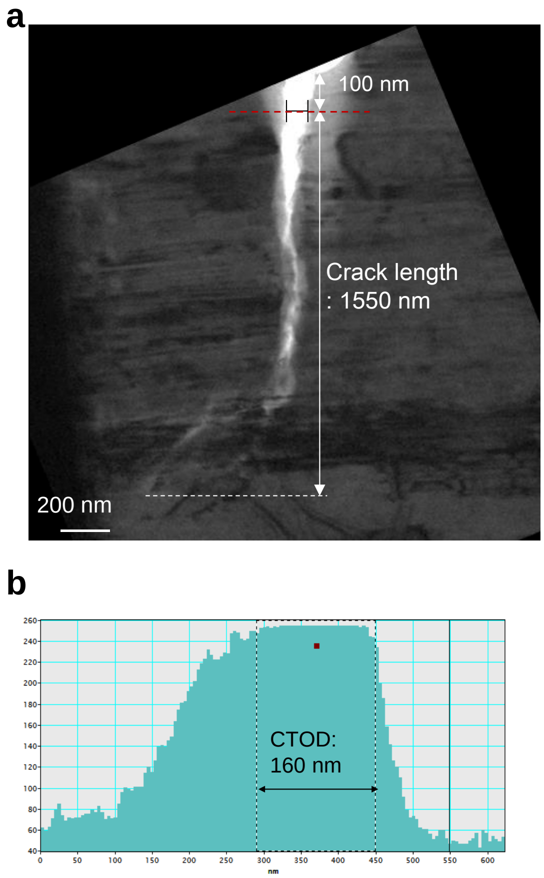

Supplementary Figure 4 | Measurement of CTOD and crack length. (a) A snapshot TEM image (captured from Supplementary Movie 1) illustrating how the CTOD and crack length are defined and measured on TEM images. (**b**) Intensity profile obtained along the red dotted line in **a** for the measurement of CTOD.

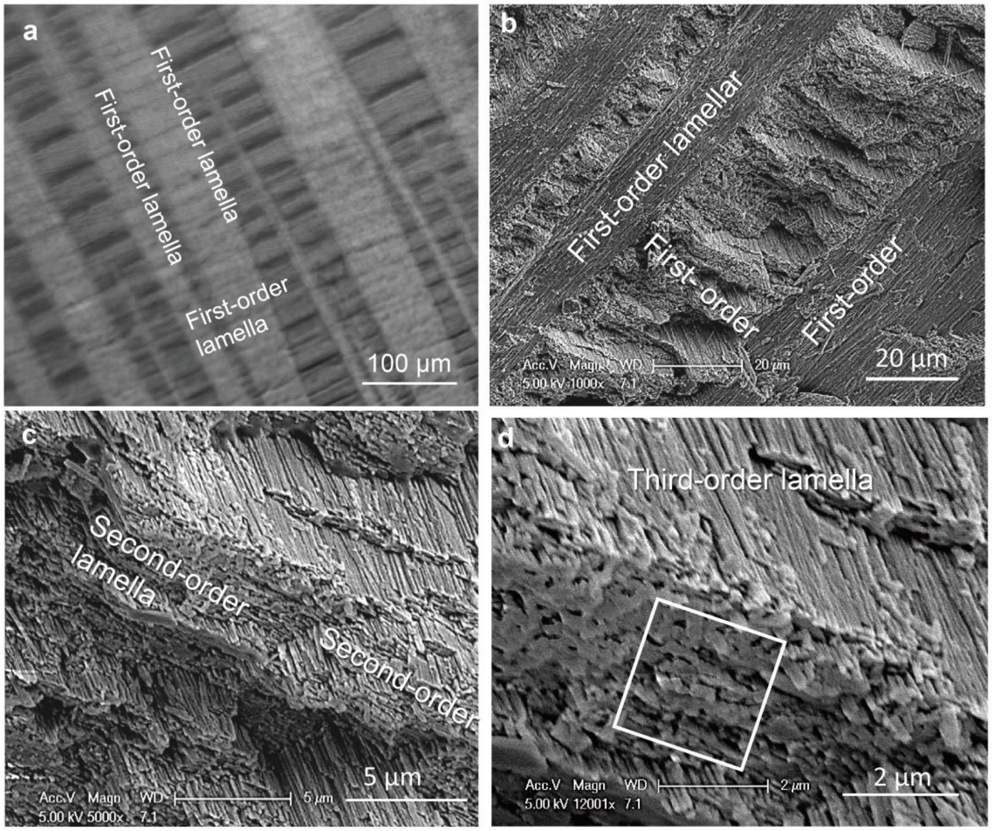

Supplementary Figure 5 | Structural hierarchy of *Strombus gigas* conch. a, Optical microscope image of a polished surface showing the crossed-lamellar stacking of first-order lamellae in the middle layer. **b**, SEM image of a first-order lamellae showing the same stacking in **a**. **c**, SEM image showing the stacking of second-order lamellae which consist of a bundle of third-order lamellae. **d**, SEM image showing a stick shape of the third-order lamellae in a second-order lamella.

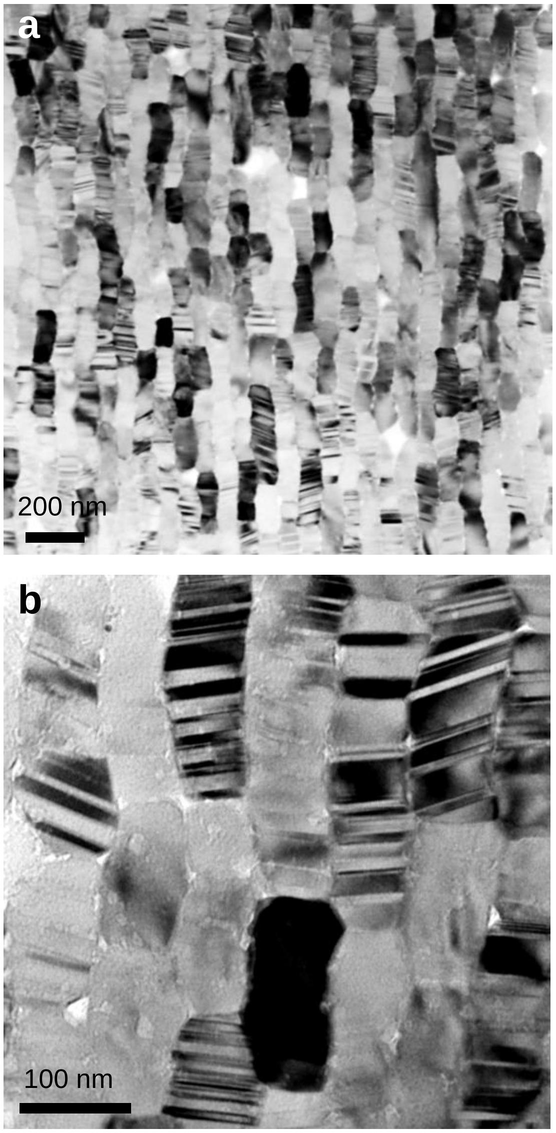

Supplementary Figure 6 | TEM end-face view of third-order lamellae. a, TEM image of the end-face of third-order lamellae. **b**, Magnified TEM image showing the nanotwins in each third-order lamella.

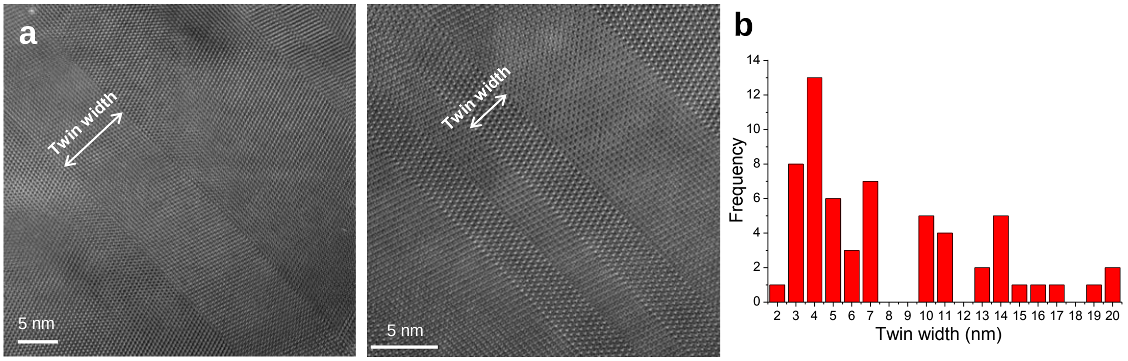

Supplementary Figure 7 | Size distribution of nanotwins in *Strombus gigas* conch shell from HRTEM images. a, The twin width, characterized by the inter-TB spacing, was measured accurately on HRTEM images taken at different locations of the third-order lamellae. HRTEM images of nanoscale twins in a third-order lamellar of *Strombus gigas* conch shell. The HRTEM images were taken using a high voltage electron microscope operated at 1.25 MV. **b**, Distribution of twin width of 60 nanoscale twins. The average width is measured to be about 8 nm with a standard deviation of 5 nm.

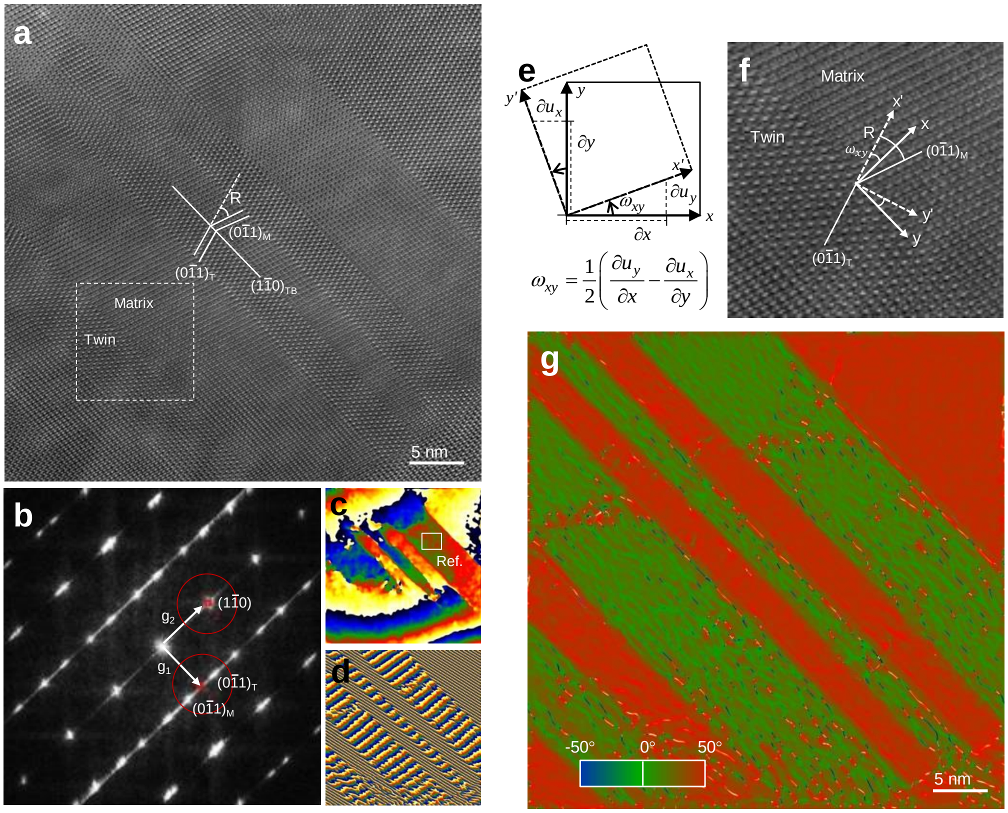

Supplementary Figure 8 | Mapping of the rotation angle of lattice planes across TBs by GPA of HRTEM image. a, A HRTEM image of nanotwinned aragonite from *Strombus gigas* conch shell. "*R*" is defined as the angle between $(0\bar{1}1)_T$ and $(0\bar{1}1)_M$ planes in the twin and matrix, respectively. **b**, Fourier transformed pattern of the HRTEM image, and selection of two reciprocal lattice vectors, $\mathbf{g_1}$ and $\mathbf{g_2}$ with an annular mask (red circles). **c-d**, Geometric phase maps of $\mathbf{g_1}$ and $\mathbf{g_2}$ vectors, respectively. The reference area is marked by the white box. **e**, Definition of rotation angle ω_{xy}, which is the direct output of GPA. **f**, Enlarged view of the white dotted box in **a**. *x*-axis is set to be perpendicular to the TBs. The definition of rotation angle "*R*" is given and compared with ω_{xy}. **g**, The calculated map of rotation angle *R*.

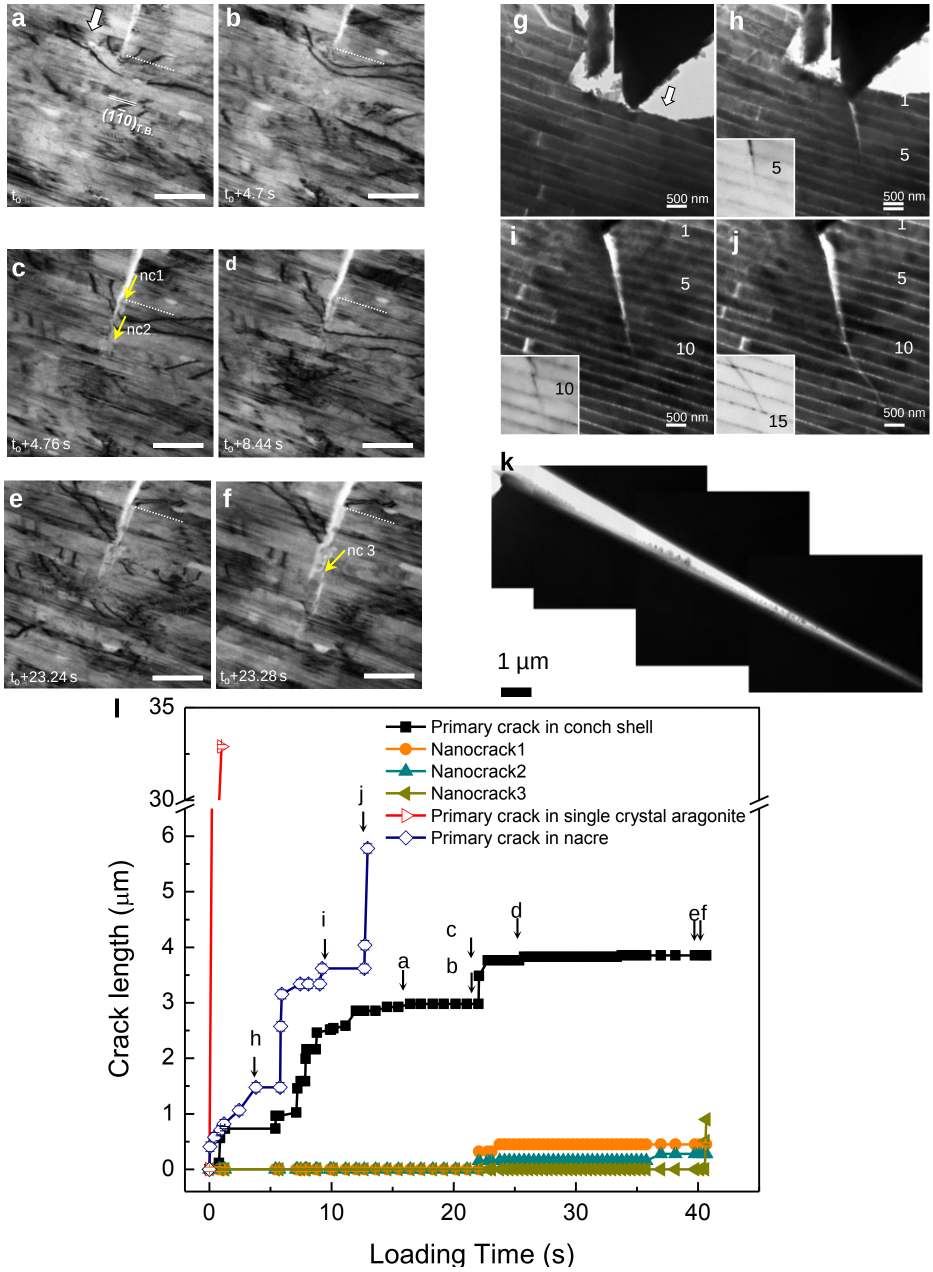

Supplementary Figure 9 | Crack propagation paths in the conch shell, nacre and single-crystalline aragonite. a, Formation of a primary crack perpendicular to TBs in the conch shell. The white arrow indicates the loading direction. The white dotted line is used to track the crack propagation. **b**, Temporary arrest of the primary crack by multiple TBs. **c**, Subsequent propagation of the primary crack through TBs accompanied by the nucleation of nanocracks (yellow arrows) near the primary crack. **d-e**, Permanent blocking of the primary crack by multiple TBs. **f**, Nucleation and propagation of a new nanocrack ahead of the primary crack tip. **g-j**, A sequence of TEM images showing crack propagation in a twin-free biogenic aragonite of nacre. The aragonite plates are numbered from the loading surface. A magnification of the crack tip in inverted color is inserted in **i** and **j**. **k**, TEM image of crack propagation in single-crystalline aragonite. **l**, Plot of the crack propagation distance vs. loading time measured in various aragonite-based crystals.

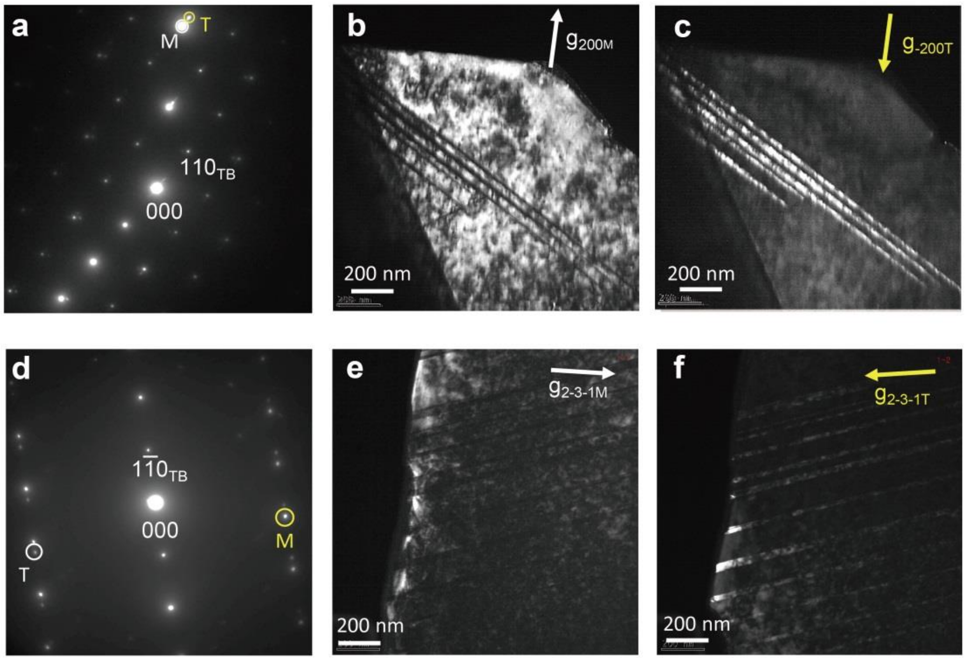

Supplementary Figure 10 | Deformation twins in aragonite single crystal. Deformation twinning pronouncedly occurred in aragonite single crystals during the CTOD measurement testing (also see Supplementary Movies 2 and 3). **a**, Selected area electron diffraction pattern of aragonite crystal containing the (110) twins. **b-c,** Dark-field TEM images formed by (**b**) the matrix reflection (denoted by M) and (**c**) the twin reflection (denoted by T) in **a**. **d**, Selected area electron diffraction pattern of aragonite crystal containing the $(1\bar{1}0)$ twinning. **e-f,** Dark-field TEM images formed by (**e**) the matrix reflection (denoted by M) and (**f**) the twin reflection (denoted by T) in **d**. Supplementary Figure 10 shows typical deformation twins and their electron diffraction patterns, indicating a common {110} deformation twinning mode in aragonite. Profuse deformation nanotwins nucleate on the (110) plane from the notch tip and other stress concentration sites, as shown in **b-c**. Some deformation nanotwins on the $(1\bar{1}0)$ plane are also shown in **d-f**.

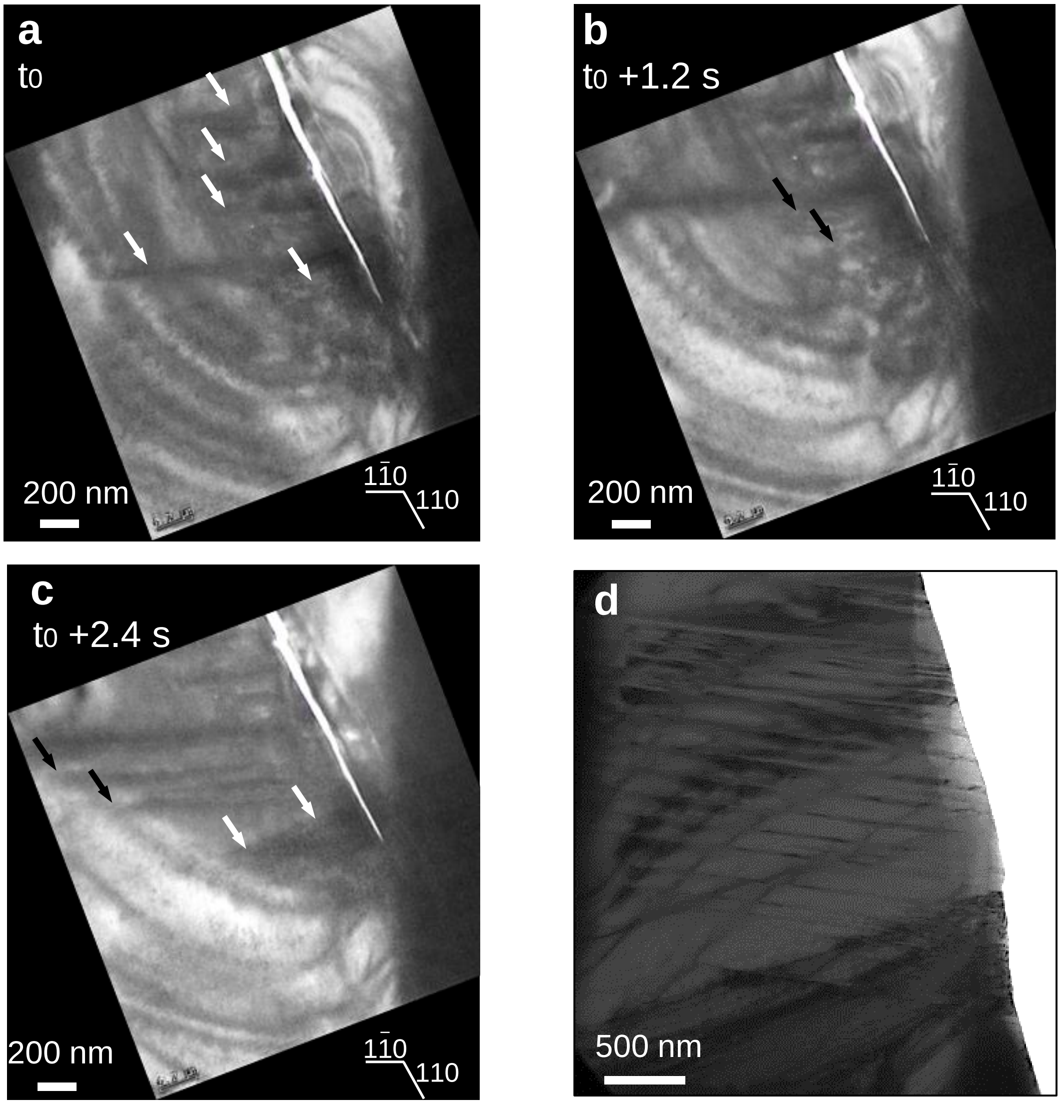

Supplementary Figure 11 | Deformation twinning induced by crack tip stress fields in aragonite single crystal. A sequence of snapshots from in situ TEM movie of Aragonite C (Supplementary Movie 3) shows that twins are nucleated at the crack tip to release the stress and then extend away from the crack tip. Although such deformation twinning can contribute to energy dissipation to some extent, these deformation twins cannot effectively hinder crack propagation. **a-c**, Snapshots from dark-field TEM movie showing ($1\bar{1}0$) deformation twinning in aragonite (Supplementary Movie 3). White arrows in **a** indicate deformation twins nucleated while the main crack propagates. Black arrows in **b**, **c** indicate the extension of deformation twins after nucleation. White arrows in **c** point newly nucleated deformation twins near the crack tip. **d**, TEM image taken after the mechanical testing. Nanoscale deformation twins formed during the crack propagation in aragonite are clearly observed.

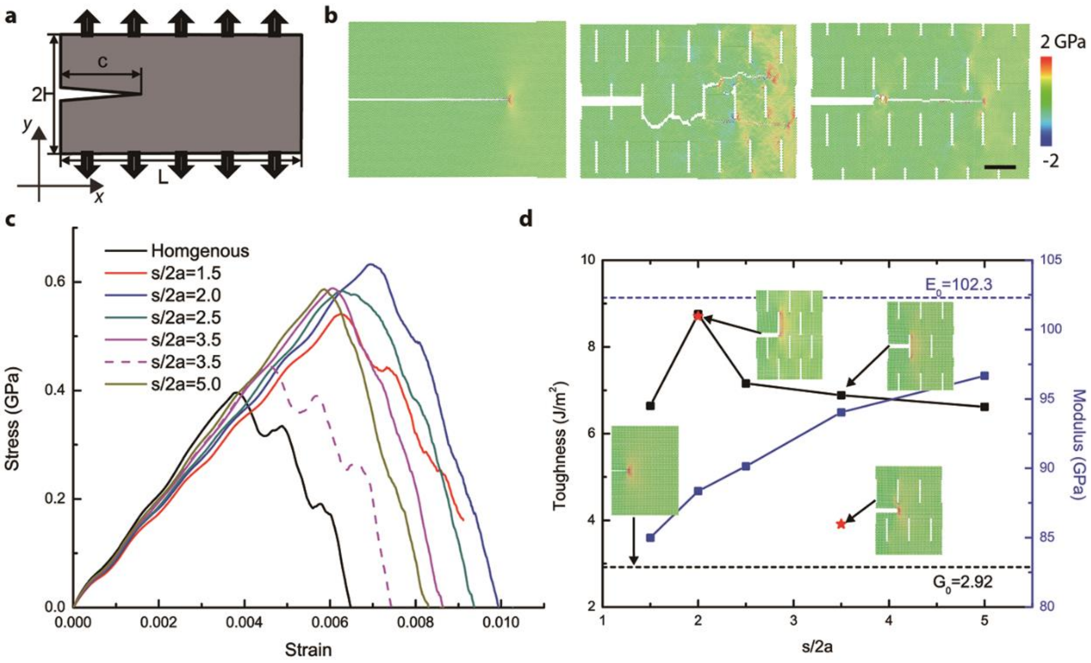

Supplementary Figure 12 | Coarse-grained modeling on toughening by *y*-nanocrack array. a, Schematic illustration of the simulated strip with an edge crack. **b,** Crack propagation paths in the homogenous material (left) and those with two typical *y*-nanocrack patterns (middle, right). Color represents the value of stress component σ_{yy}. **c,** Stress-strain curves of finite strips with different nanocrack periodicities. The two curves with the same color (purple, $s/2a$ = 3.5) were calculated for the main crack located at different locations with respect to the nanocrack array; the solid purple line represents the case when the main crack directly intersects a nanocrack (middle figure in **b**), while the dashed purple line is for the main crack located between two neighboring nanocracks (right figure in **b**). **d,** The toughness and elastic modulus as a function of the normalized crack periodicity, defined as *s/2a*. The crack spacing *d* and length 2*a* are both fixed at 200 nm during the simulations. Red star represents the toughness of the structure with a shift in the relative position of the main crack with respect to nanocracks. Scale bar: 200 nm.

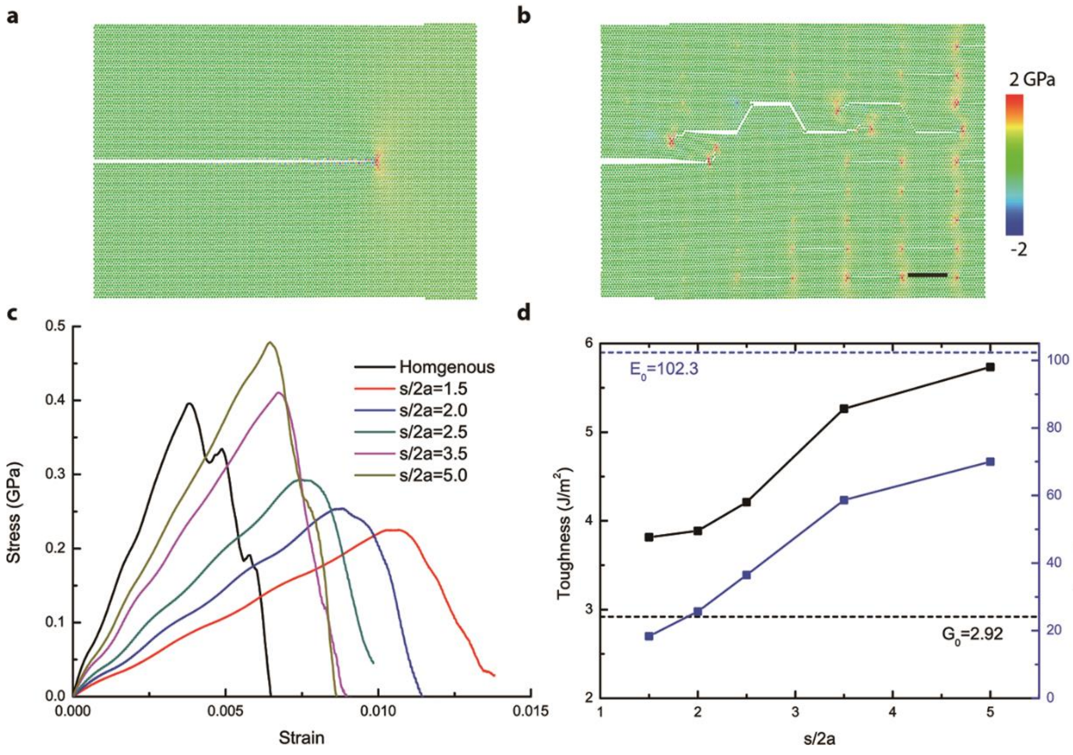

Supplementary Figure 13 | Coarse-grained modeling on toughening by *x*-nanocrack array. a, Crack propagation path in the homogenous material. **b,** Typical crack propagation path in a *x*-nanocrack-patterned structure. The color in **a** and **b** represents the value of stress component σ_{yy}. **c,** Stress-strain curves for materials with different nanocrack periodicities. **d,** The toughness (black symbols) and elastic modulus (blue) as functions of the normalized crack periodicity $s/2a$. The crack spacing d and length $2a$ are both fixed at 200 nm during the simulations. Scale bar: 200 nm.

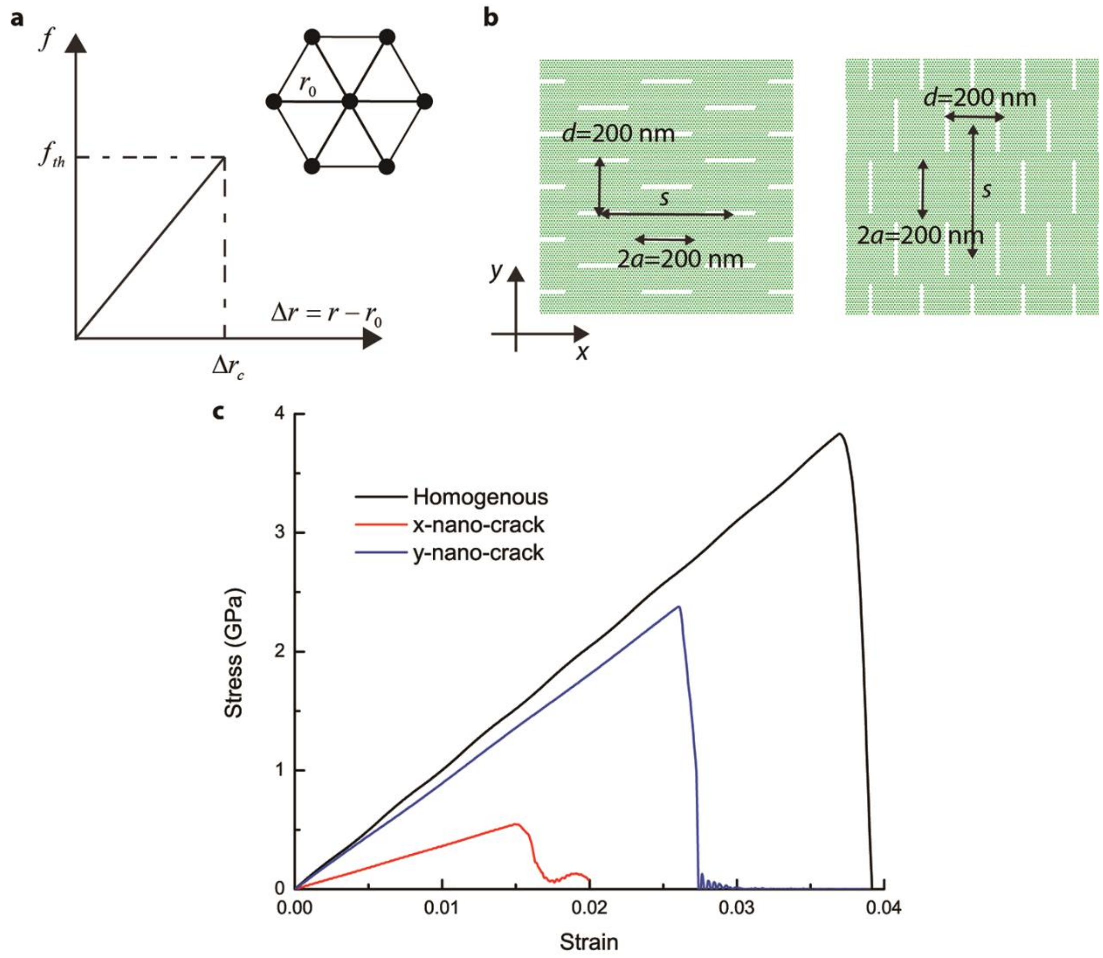

Supplementary Figure 14 | Coarse-grained modeling of aragonite with arrays of nanocracks. a, A triangular lattice model of aragonite with a linear force-extension relationship for each bond. **b**, Materials with *x*- and *y*-nanocrack patterns, corresponding to nanocracks parallel to the *x* and *y* directions, respectively. **c**, Stress-strain curves of a perfect material and those with different nanocrack patterns.

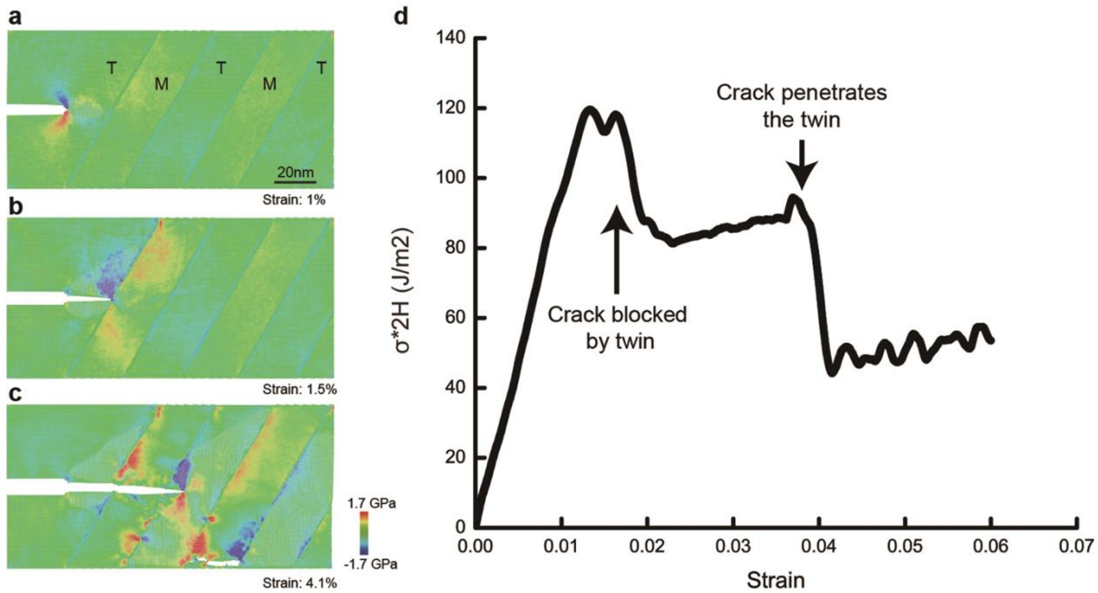

Supplementary Figure 15 | Atomistic simulations of crack propagation in nanotwinned aragonite with TB spacing of 20 nm. a-c, A sequence of snapshots of crack propagation in a nanotwinned aragonite sample with inclined TBs. **d,** Stress-strain curves of nanotwinned aragonite with an edge crack.

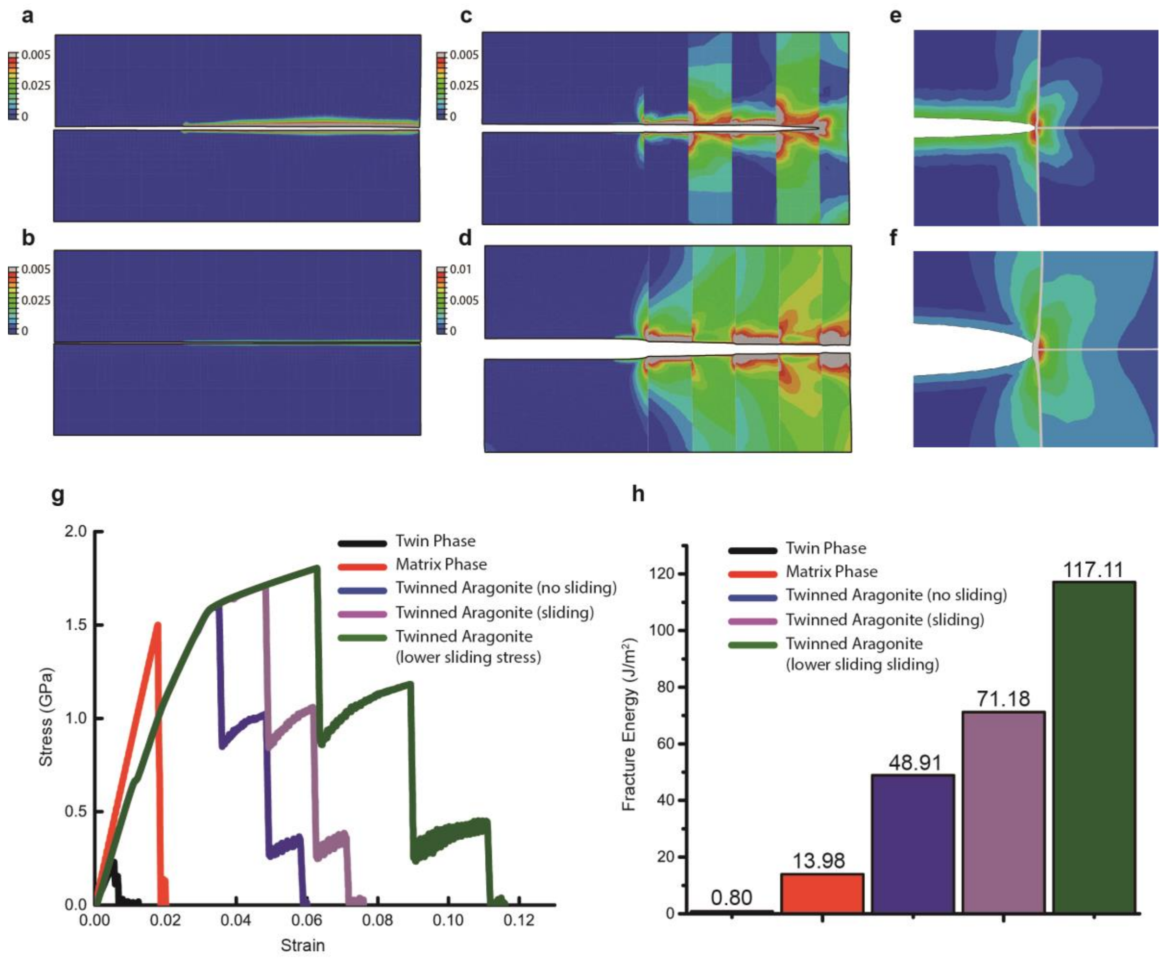

Supplementary Figure 16 | FEM modelling of twinned aragonite with TB sliding and crack tip blunting effects. a-b, Crack propagation in single twin and matrix phases. Very limited plastic strain observed in these two single-crystalline samples. **c-d,** Crack propagation in twinned aragonite without and with boundary sliding. **e-f,** Configurations of the crack tip without and with boundary sliding. **g,** Stress-strain curves of different modelling cases. **h,** Fracture energy of different modelling cases. Colors in **a-f** represent the effective plastic strain.

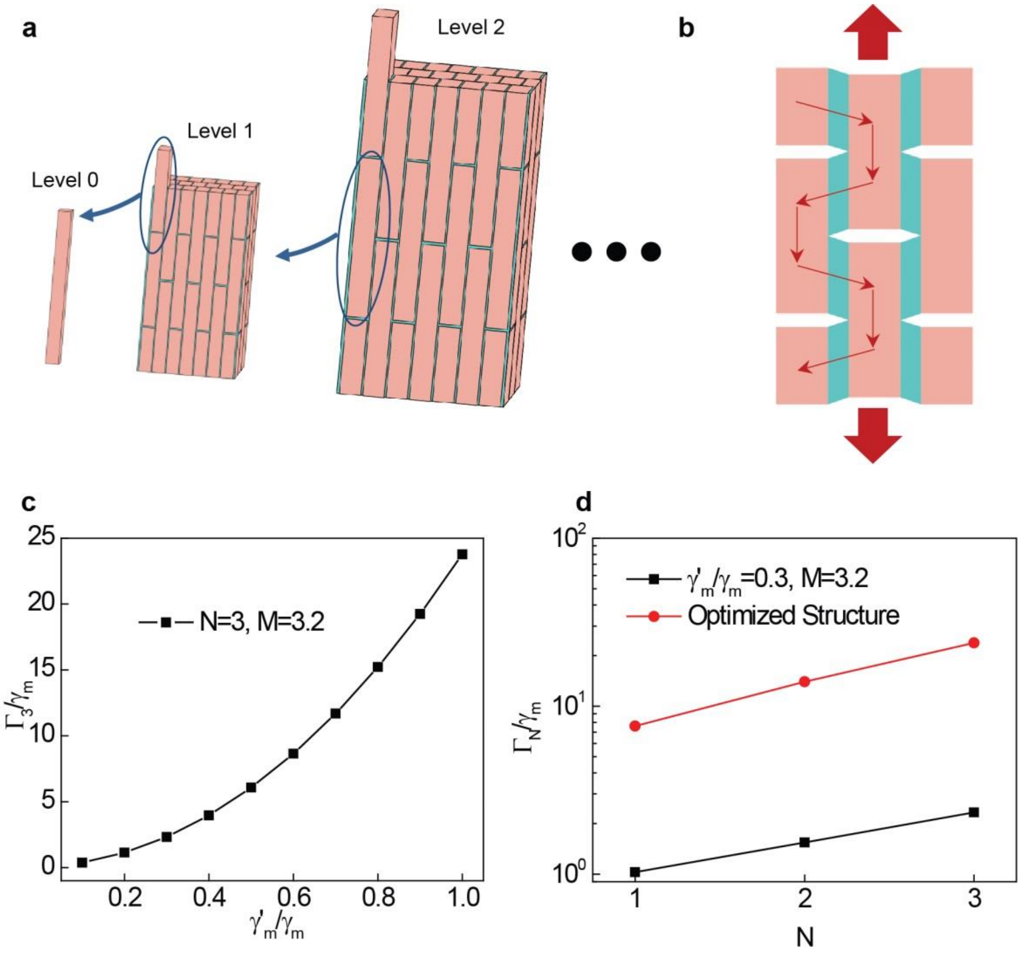

Supplementary Figure 17 | Model of quasi-self-similar hierarchical structure and contribution of the lowest-level structure to the overall toughness of a hierarchical material. a, Schematic illustration of the quasi-self-similar hierarchical model. Each level consists of staggered hard mineral inclusions (pink) embedded in a soft organic matrix (green). **b,** A tension-shear chain model showing mineral inclusions carry load primarily by tension and the organic matrix by shear. **c,** Toughness of the 3-level hierarchical structure as a function of the lowest-level mineral fracture energy $2\gamma'_m$. **d**, Variations of toughness with number of hierarchical levels N. Here γ_m denotes the intrinsic fracture energy of mineral, which is about 1 J/m^2. M represents the strain hardening index of the soft matrix.

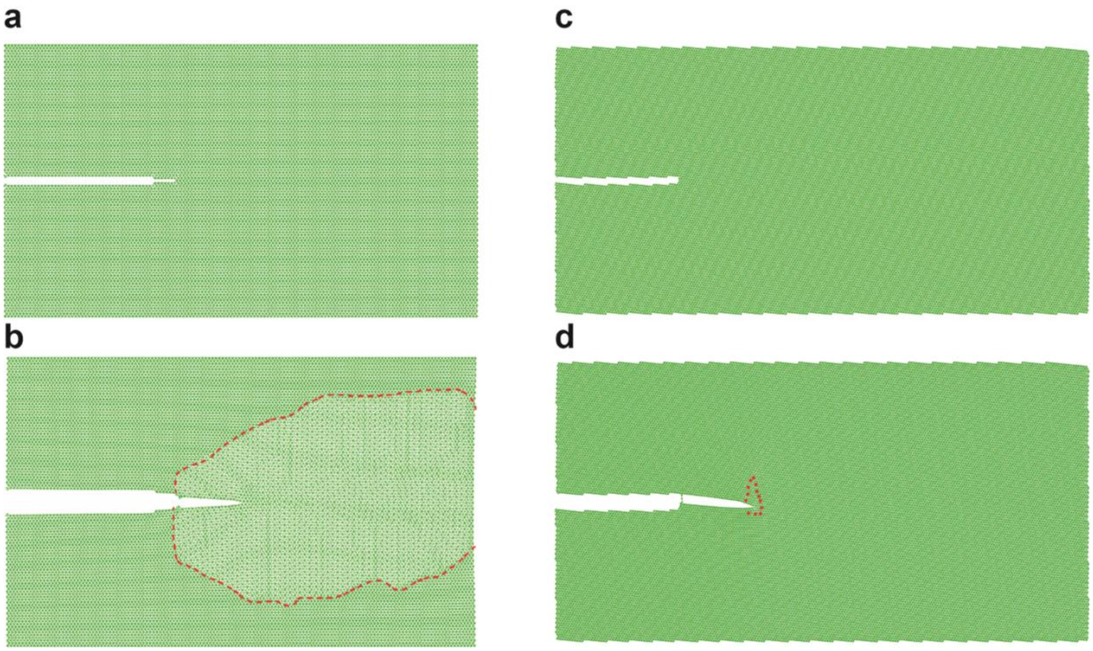

Supplementary Figure 18 | Crack propagation in twin-free aragonites with T- and M-orientations. a-b, Atomic configurations of a twin-free aragonite sample with T-orientation. The crack propagates straight ahead with structural transformation ahead of the crack tip. **c-d,** Atomic configurations of a twin-free aragonite sample with M-orientation. The crack is slightly deflected during propagation. All transformed regions are indicated by red dashed lines.

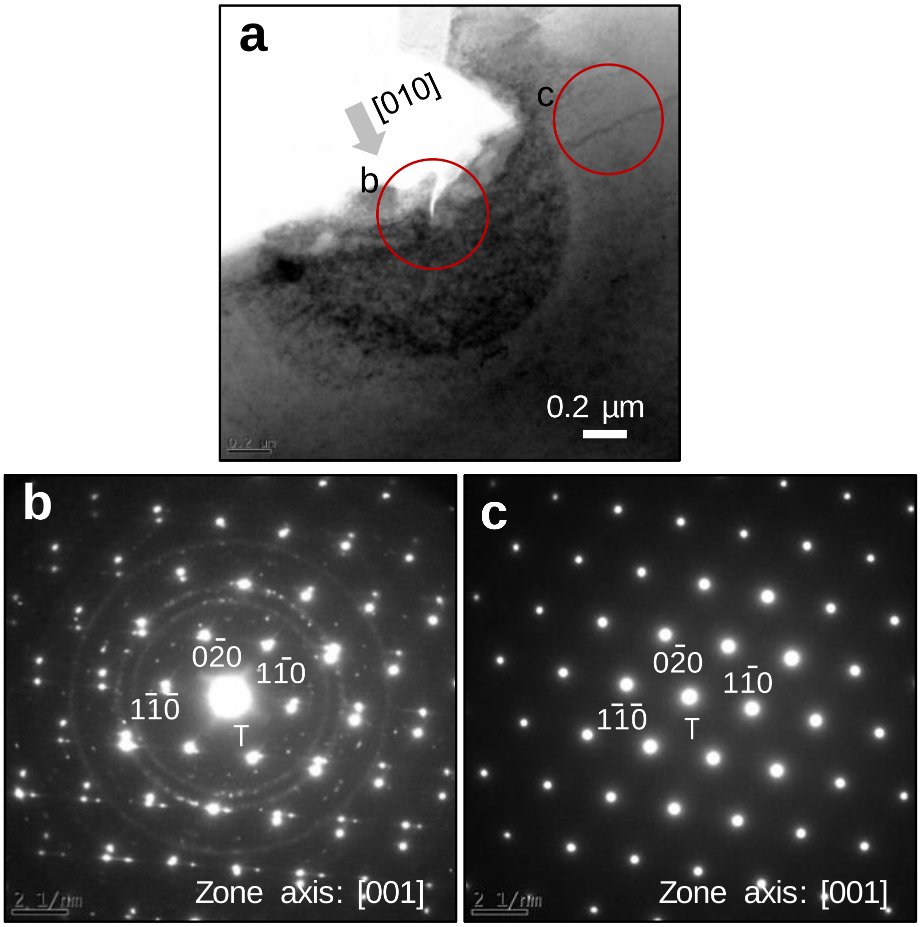

Supplementary Figure 19 | Phase transformation of aragonite in nanoindentation. a, A low-magnification TEM image of single-crystalline aragonite after *in situ* TEM nanoindentation along the [010] direction. **b-c**, Electron diffraction patterns along [001] zone axis of the circled regions b and c in **a**. The results suggest the formation of nanocrystalline phase in a hemispherical zone beneath the indenter.

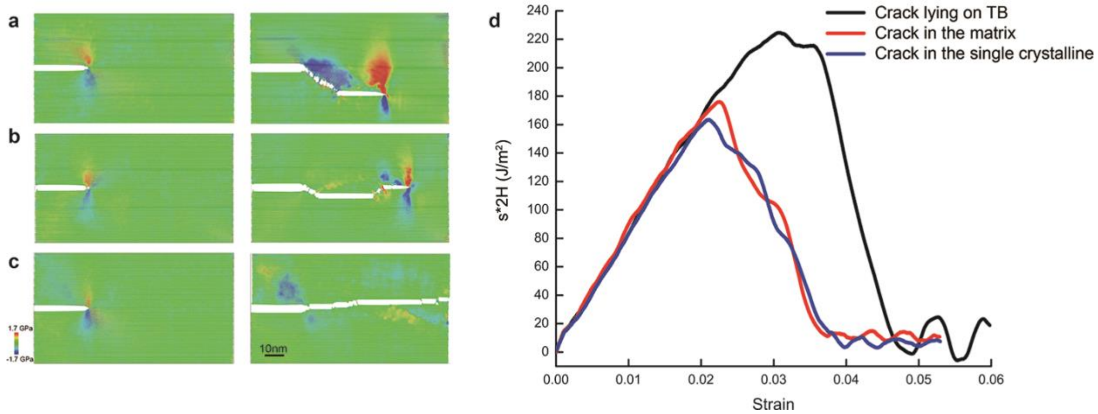

Supplementary Figure 20 | Atomistic simulations of crack propagation parallel to TBs in a nanotwinned aragonite sample. a, Crack propagation in nanotwinned aragonite with a crack initially lying on a TB. **b,** Crack propagation in nanotwinned aragonite with a crack initially parallel to TB in the matrix domain. **c,** Crack propagation in single-crystalline aragonite. Here the initial crack is parallel to [110]. Crack deflection is clearly observed in **a-c**, where the color represents the in-plane shear stress. **d,** Stress-strain curves of samples in (**a-c**).

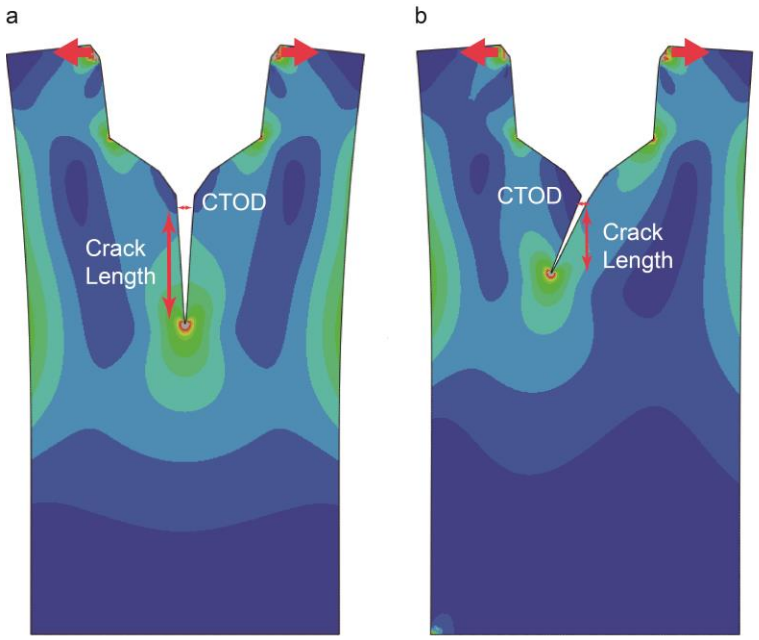

Supplementary Figure 21 | FEM modelling of CTOD experiments. a-b, FEM models of the notched nanotwinned aragonite sample **(a)** and single-crystalline aragonite sample **(b)**. The sample geometries are the same as those in the experiment, and the red arrows indicate loading direction.

Supplementary Table 1. Ratio of the magnitude of reciprocal lattice vectors in aragonite, calcite, and new phase formed by nanoindentation.

g_n/g_1	Aragonite	Calcite	New Phase (Formed by nano-indentation)
g_1/g_1	1	1	1
g_2/g_1	1.106	1.27	1.206
g_3/g_1	1.169	1.356	1.398
g_4/g_1	1.372	1.545	1.971
g_5/g_1	1.423	1.687	
g_6/g_1	1.623	1.84	
* *n* denotes a sequence of ring, counting from the shortest reciprocal lattice vector.			

Supplementary Note 1 | Structural hierarchy of *Strombus gigas* conch shell

A shell of *Strombus gigas* is mainly divided into three layers, i.e. the outer, middle and inner layers. Each layer has a three-level hierarchical microstructure, the so called crossed-lamellae structure, containing first-, second- and third-order lamellae (Supplementary Figure 5). The first-order lamella, with width of 5-60 μm and height of ~2 mm, is oriented at 90º with respect to the adjacent first-order lamellae (Supplementary Figure 5a-b). Each first-order lamella consists of bundles of second-order lamellae with width of 5-60 μm and height of 5-30 μm (Supplementary Figure 5c). These second-order lamellae are built up from parallel-stacked third-order lamellae which are ~100 nm thick and 100-400 nm long (Supplementary Figure 5d). The third-order lamellae are made of nanotwinned aragonite platelets. Supplementary Figure 6a-b show TEM images of end-faces of third-order lamellae within the dotted square in Supplementary Figure 5d. The stripe contrasts in Supplementary Figure 6 indicate high density of nanotwins in the third-order lamellae. Each first-, second- and third-order lamella is tightly interlocked and surrounded by proteinaceous matrix.

Supplementary Note 2 | Measurement of twin-induced lattice rotation

Geometric phase analysis (GPA) of HRTEM images was used to quantitatively assess the crystallographic rotation of a lattice plane (indicated by '*R*' in Supplementary Figure 8a) across the TBs. The measurement is based on the routine angle measurement tool provided by GPA package[15]. In the calculation, the two reciprocal lattice vectors in the Fourier transformed pattern of a HRTEM image, $\mathbf{g_1}$ corresponding to the midpoint between $(0\bar{1}1)_{\mathrm{T}}$ and $(0\bar{1}1)_{\mathrm{M}}$, and $\mathbf{g_2} = 1\bar{1}0$, are selected with an annular mask, where $\mathbf{g_1}$ contains the two reflections related to the twin relationship and $\mathbf{g_2}$ corresponds to the twin plane (Supplementary Figure 8b). The geometric phase of $\mathbf{g_1}$ and $\mathbf{g_2}$ are obtained (Supplementary Figures 8c-d) and used to calculate the angle (ω_{xy}) of corresponding lattice planes with respect to the reference frames (x and y), according to the following expression,

$$\omega_{xy} = \frac{1}{2}\left(\frac{\partial u_y}{\partial x} - \frac{\partial u_x}{\partial y}\right), \tag{1}$$

where u_x is the displacement along the x-axis and u_y along the y-axis (Supplementary Figure 8e). When the x-axis is selected to be perpendicular to the TBs (Supplementary Figure 8f), the value of ω_{xy} is calculated as 18º. According to the definition of R, R is twice of ω_{xy}. Hence, the misorientation between twin and matrix is 36º, as shown in Supplementary Figure 8g.

Supplementary Note 3 | *In situ* TEM observations of crack propagation paths in conch shell, nacre and single-crystalline aragonite

In situ TEM nanoindentation was performed on three different aragonite-based crystals, namely, nanotwinned aragonite samples from *Strombus gigas* conch shell, layer-by-layer stacked aragonite from nacre, and single crystalline aragonite as a reference. Note that the *Strombus gigas* conch contains 99 wt% aragonite and 1 wt% protein, while the nacre has 95 wt% aragonite and 5 wt% protein. Except the difference in volume/weight fractions of mineral and organics, the most remarkable discrepancy is that the aragonite in the conch shell contains a high density of nanoscale growth twins, whereas that in the nacre is single-crystalline and twin-free. The TEM specimens were prepared by mechanical wedge polishing as shown in Supplementary Figure 1 and tested following the method described in Supplementary Figure 2. Supplementary Movie 5 shows the original *in situ* TEM movies along with quantitative measurements of crack length vs. loading time.

A sequence of TEM images in Supplementary Figures 9a-f captures a typical process of crack propagation in the nanotwinned aragonite from conch shell, where a main crack is seen to initiate and then propagate nearly perpendicular to the TBs. It is observed from Supplementary Figures 9 a-b and d-e that crack propagation is effectively blocked by multiple TBs, as evidenced by two clear plateaus in the crack advancing curve in Supplementary Figure 9l. In the layer-by-layer stacked twin-free aragonite from nacre, compared with the nanotwinned aragonite from conch shell, the main crack easily penetrates through individual aragonite plates without hindrance, and are only occasionally deflected at the organic interfaces between adjacent plates (Supplementary Figures 9h-j), as also shown by the plateaus in the curve shown in Supplementary Figure 9l. This result supports the widely held belief that the toughness of nacre mainly derives from the soft organic layer between aragonite platelets. In contrast, in the aragonite single crystal, once the main crack initiates in a brittle manner along the cleavage planes of (110) and (010) in a zigzag pattern, it propagates all the way through the crystal without any hindrance (Supplementary Figure 9k).

Supplementary Note 4 | Nanocrack toughening

Detailed TEM observations reveal that a few nanocracks are formed in the vicinity of the primary crack tip with a distinct pattern (Figs. 4a-d; Supplementary Movie 4). To investigate the effect of the observed nanocrack patterns on the toughness, we carried out a series of coarse grained simulations of samples with crack period s varying from 300 nm to 1000 nm, while the crack spacing d and half-length a are kept fixed. Two patterns of nanocracks were considered in the simulations, one perpendicular (referred to y-nanocracks) and the other parallel (x-nanocracks) to the primary crack (see Supplementary Figure 14b). Our simulation indicates that the toughness of the structure with y-nanocracks, even with a lower elastic modulus, can be enhanced by 2.2–3 times compared with the homogenous case, which is consistent with previous theoretical predictions[17-20]. More interestingly, an optimum crack pattern identified from the current simulations (s=400 nm, a=100 nm and d=200 nm), as shown in Supplementary Figure

12d, is very close to that observed in the current *in situ* experiments of crack propagation in *Strombus gigas* conch shell (Fig. 4). It is also noticed that a shift of the relative position of main crack with respect to nanocracks can dramatically reduce the toughness for large crack periodicity. On the other hand, the toughness is insensitive to the main crack position for small crack periodicity. For the structure with *x*-nanocrack, the toughness monotonously increases with the normalized crack period, as indicated by Supplementary Figure 13d.

Supplementary Note 5 | Atomistic simulations of crack propagation in nanotwinned aragonite with TB spacing of 20 nm

In order to check the effect of twin width on fracture toughness, we repeated the atomistic simulation for nanotwinned aragonite with a larger TB spacing of 20 nm. Such system includes about 1.2 million atoms in total. Supplementary Figures 15a-c shows a sequence of snapshots of crack propagation in the simulated sample with inclined TBs. When the crack approaches TBs, it is arrested due to the blocking effect of TBs for some time, which is essentially similar to the simulations with the TB spacing of 10 nm (Figs. 5c-d in the main text). The in-plane shear stress contours in Supplementary Figures 15b-c also suggest the delocalization of deformation due to the presence of twins. By integrating the stress-strain curve in Supplementary Figure 15d until failure and then multiply by strip width, we obtain a fracture energy of about 3.3 J/m^2, which is comparable but smaller than the value of 5.13 J/m^2 when TB spacing is 10 nm. Note that the average twin size of *Strombus gigas* conch shell is measured to be 8 nm. This suggests that a high density of nanoscale twins is necessary to induce sufficient toughening in the material.

Supplementary Note 6 | Structural transformation in aragonite under stress: *in situ* TEM nanoindentation and MD simulations

It was observed in MD simulations that a structural transformation occurs ahead of the a crack tip in aragonite before it starts to propagate during loading. In our simulations of crack propagation in single-crystalline aragonite, it was found that the structural transformation easily occurs in the samples with T-orientation (Supplementary Figures 18a-b). Moreover, the transformed area in samples with T-orientation is much larger than that in samples with M-orientation (Supplementary Figures 18c-d), implying a strong anisotropy effect on transformation in single-crystalline aragonite. In a twin-free aragonite crystal with M-orientation, the transformed area around the crack tip is very small (Supplementary Figure 18d), so the crack essentially cleaves ahead along the cleavage plane.

Supplementary Figures 19b and 19c show the electron diffraction patterns along the [001] zone axis of the indent and surrounding regions, respectively, shown in Supplementary Figure 19a. The comparison between Supplementary Figures 19b and 19c

reveals that, while the surrounding region (red circle c in Supplementary Figure 19a) remains single-crystalline, new ring patterns and streaks in the reflections appear in the indent region (red circle b in Supplementary Figure 19a). The diffraction rings which appear at different *d*-spacings from those of both aragonite and calcite (Supplementary Table 1) with fine and non-diffusive pattern indicate the formation of a new nanocrystalline phase in random orientation. Similar phenomena were also reported in the biogenic calcite from *P. Placenta* shell under nanoindentation[21] and the aragonite of *Busycon carica* shell under electron beam irradiation[22].

Supplementary Note 7 | Atomistic simulations of crack propagation parallel to TBs

To investigate the influence of TBs orientation on crack propagation, we also simulated crack propagation parallel to TBs in a nanotwinned aragonite sample, and compared the results with the cases of single-crystalline aragonites, as well as nanotwinned aragonites with the crack inclined to TBs. Supplementary Figures 20a-c show some typical snapshots of crack propagation in three different samples. According to the stress-strain curves in Supplementary Figure 20d, we calculated the effective toughness using Eq. (15). For nanotwinned aragonite, the toughness in the case of a crack initially lying on a TB is about 3.66 J/m^2. If the crack lies initially in the matrix domain (i.e. between two neighboring TBs) parallel to the TBs, the fracture toughness is about 2.08 J/m^2. Both values are smaller than that of 5.13 J m^{-2} in the case of crack propagating inclined to the TBs (about 58°), but substantially larger than those (0.55 J/m^2 and 0.28 J/m^2) in single-crystalline aragonite samples with initial crack parallel to [100]. These results indicate that the presence of TBs significantly increase the fracture toughness of aragonite, and the relative orientation between TBs and the crack has significant effect on fracture toughness. We also noted that in single-crystalline aragonite with crack propagation parallel to [110] (i.e. TB orientation), the fracture toughness is about 1.67 J/m^2, which is larger than those (0.55 J/m^2 and 0.28 J/m^2) for crack propagation parallel to [100]. This confirms the existence of distinct cleavage directions in aragonite crystal, and the strong anisotropy in fracture toughness may have played an important role in the evolution of hierarchical structures of the conch shell.

Supplementary Note 8 | Self-similar model for optimized hierarchy of conch shell

To understand how toughening at the level of nanotwinner aragonite in third order lamellae affects the overall toughness of conch shell, we adopt a quasi-self-similar hierarchical model[23-25]. In this model, each hierarchical level has a similar structure of staggered hard plates embedded in a soft organic matrix, and the structure of each level serves as hard plates at the next level, as illustrated by Supplementary Figure 17a. Under uniaxial tensile stress, the hard plates primarily carry load by tension while the protein matrix transfers load to neighboring plates by shear[23] (Supplementary Figure 17b). Under the assumption that such hierarchical structure has evolved into optimized stiffness and toughness, a flaw-tolerance criterion is used to determine the characteristic width h_n of hard inclusion at the n-th level as,

$$h_n \le \frac{E_n \Gamma_n}{S_n^2},\ n = 0, 1, 2, ..., N \tag{2}$$

where E_n, S_n, Γ_n denote the Young's modulus, strength and fracture energy at the n-th level, respectively. At the lowest level (i.e. n=0), the basic properties of mineral are defined as,

$$E_0 = E_m,\ S_0 = \sigma_m,\ \Gamma_0 = 2\gamma_m \tag{3}$$

where E_m is the Young's modulus, σ_m the theoretical strength and γ_m the fracture surface energy of mineral.

In the optimized hierarchical structure, the hard inclusion and soft matrix simultaneously reach their failure strengths. Accordingly, the aspect ratio $\rho_n = l_n / h_n$ of hard inclusion at the n-th level can be determined as[23-25],

$$\rho_n = \frac{S_n}{\tau_n^p},\ n = 0, 1, 2, ..., N-1 \tag{4}$$

where S_n is the tensile strength of the inclusion and τ_n^p represents the failure stress of the protein matrix. The Young's modulus at the n-th level is expressed as[23-25],

$$E_n = \left[\frac{4(1-\varphi_{n-1})}{G_{n-1}^p \varphi_{n-1}^2 \rho_{n-1}^2} + \frac{1}{\varphi_{n-1} E_{n-1}} \right]^{-1} \tag{5}$$

The strength at the n-th level is,

$$S_n = \min(\frac{1}{2}\varphi_{n-1}\rho_{n-1}\tau_{n-1}^p, \frac{1}{2}\varphi_{n-1}S_{n-1}) \tag{6}$$

where φ_{n-1} and ρ_{n-1} denote the volume fraction and aspect ratio of the hard inclusions, while G_{n-1}^p and τ_{n-1}^p represent the shear modulus and strength of the soft matrix, respectively. The fracture energy at the n-th level is estimated as[23],

$$\Gamma_n = (1-\varphi_{n-1}) h_{n-1} \rho_{n-1} \int \tau d\varepsilon + \varphi_{n-1} 2\gamma_m,\ n = 1, 2, ..., N-1 \tag{7}$$

We here assume that the plastic dissipation of protein matrix obeys a power-law constitutive equation,

$$\varepsilon = K\tau^M, \text{ where } M > 1 \tag{8}$$

Thus, the fracture energy can be re-written as,

$$\Gamma_n = (1-\varphi_{n-1}) h_{n-1} \rho_{n-1} \frac{M}{1+M} K \tau^{M+1} + \varphi_{n-1} 2\gamma_m, \text{ where } \tau = \min(\tau_{n-1}^p, \frac{S_{n-1}}{\rho_{n-1}}) \tag{9}$$

For the strain hardening index M, we fit the stress-strain curves from the literature[26] and find it to be 1.6~3.2. Moreover, we assume that the soft matrix at every level have the same mechanical properties, i.e.,

$$\tau_n^p = \tau^p, G_n^p = G^p, \Theta_n^p = \Theta^p \text{ and } \Theta^p = K(\tau^p)^M \tag{10}$$

where τ^p, G^p, Θ^p represent the shear strength, shear modulus and failure shear strain of the proteinacious matrix, respectively. For the present quasi-self-similar model, Φ is taken to be about 95%, and the volume fraction of hard inclusions at each level is assumed to be identical, i.e.,

$$\varphi_n = \Phi^{1/N}, n = 0, 1, 2, ..., N-1 \tag{11}$$

Following the above equations, we can construct a quasi-self-similar hierarchical material with optimized properties. For a 3-level hierarchy like conch shell, if we substitute the following parameters into the above equations,

E_m=100 GPa, $\sigma_m=E_m/75$, γ_m=1 J/m^2, $\tau^p=\sigma_m/25$, $G^p= E_m/1000$,

Θ^p=1, Φ=0.95, M=1.6~3.2,

we can obtain the optimized material properties at each level of the hierarchy. The relevant results are shown in Supplementary Figures 17c-d.

Supplementary Methods

Design and fabrication of *in situ* TEM Specimen for CTOD measurement. The fractu re toughness in the elastic-plastic regime is determined by the *J*-integral or the crack-tip-o pening-displacement (CTOD) δ[1,2]. The *J*-integral represents the energy per unit surface ar ea required for crack propagation, while CTOD describes the opening displacement near t he crack tip. Both of them can reflect the resistance to fracture when the material undergo es plastic deformation prior to failure, and have been widely used in standard mechanical testing to determine the fracture toughness[1,2]. These two parameters are essentially equiva lent, and their relationship under the standard testing condition can be written as[1,2],

$$J = m\sigma_y\delta\,;\ \delta = \frac{J}{m\sigma_y} \qquad (12)$$

where *m* is a conversion factor depending on specimen geometry (usually in the range of 1-2), and σ_y is the yield strength of the material. In the current experiment, CTOD can be directly measured in TEM, which enables us applying standard CTOD approaches in the mechanical testing without calibration functions. Moreover, we calculate the corresponding *J* integral via FEM modeling following the same geometry and loading conditions from the CTOD experimental measurement. The relevant details are given in Methods.

To enable measurement of CTOD at nanoscale, we designed a unique TEM specimen which can be used in a nanoindentation TEM holder[1, 2]. Supplementary Figures 3a and 3b show schematic illustrations of the sample design. In Supplementary Figure 3a, the internal edges of the column heads are cut by 45˚ to secure good contact and also to reduce friction with the indenter tip (Fig. 3g). The height of the column is set to 1.3 μm to ensure adequate space for movement of the indenter tip. While the width and the thickness of the column are set at 1.5 μm and 1 μm, respectively, the thickness of the testing area of 2 μm × 3 μm is thinned down to 200 nm for electron transparency. In order to further focus stress concentration at a pre-designed notch, the corners of the testing area were cut into 30˚-inclined edges, and a notch tip was introduced at the center of the testing area.

The specimens used for *in situ* TEM CTOD measurement were prepared using focused ion beam (FIB, Helios Nano-Lab, FEI) machining techniques. The *Strombus gigas* conch shell and aragonite single crystal were first sliced and mechanically polished (Supplementary Figure 3c). A thin lamella was then lifted out of the polished surfaces and attached to a Cu grid by Pt deposition (Supplementary Figure 3d). Subsequently, an initial structuring and thinning process was performed following the design in Supplementary Figure 3b, with a beam current of 93 pA at 30 kV (Supplementary Figure 3e). Fine machining into the design in Supplementary Figure 3a was performed with a beam current of 48 pA at 30 kV (Supplementary Figure 3f). Finally, low-energy millings with beam voltages of 5 kV and 1 kV were performed to minimize Ga^{+} ion damages of the specimen surface.

The CTOD was measured and traced directly from the *in situ* TEM movies. Based on the definition of δ_5 type CTOD[3], the CTOD in this study is defined as the opening

displacement at a site which is about 100 nm away from the initial notch tip. This convention was adopted to prevent vagueness in image contrast at the notch tip and vacuum.

We used Digital Micrograph™ (Gatan) to measure the CTOD and crack extension from several key snapshots of *in situ* TEM movies, as exemplified by Supplementary Figures 4a and 4b. The CTOD is measured in the intensity profile by identifying a specific site which is about 100 nm away from the initial notch tip (Supplementary Figures 4a-b). The effective crack length is set as the projected length of the actual crack path, measured from the point where the CTOD is defined (Supplementary Figure 4a). The location of the crack tip was determined by visual inspection of the TEM images. The CTOD-R curves measured in the conch shell and aragonite single crystal are shown in Fig. 2e.

FEM modeling of crack propagation. Crack propagation in the twinned aragonite is simulated using a finite element method (FEM) (Supplementary Figure 16), where the elastic anisotropy of twin/matrix phases and the boundary sliding effect were considered.

The FEM simulations were performed with ABAQUS/Explicit on notched samples consisting of alternating twin and matrix phases. Both phases were modeled using 4-node CPS4R elements with anisotropic elasticity and isotropic hardening plasticity with different crystallographic orientations. The crack is modeled by cohesive elements with different parameters in the twin and matrix phases. To model crack blunting effects due to TB sliding, the TBs are also modeled by cohesive elements. Both crack and TBs are modelled by COH2D4 cohesive elements in ABAQUS, for which typical linear triangular traction-separation law and maximum nominal stress criterion are adopted. To model TB sliding, the critical cohesive stress in the normal direction are set high enough to ensure very small normal strain, along with a slight softening in shear stress during sliding. The cohesive stress in the tangential direction corresponds to the critical sliding stress for TBs. A constant velocity loading is applied on the top surface of the sample and the strain rate is set low enough to ensure a quasi-static process.

Supplementary Figures 16a-f shows some representative snapshots from the FEM simulations for different samples under different sliding conditions. In the cases of single-crystalline twin and matrix phases, the crack rapidly runs through the overall sample along the given cleavage surface (Supplementary Figures 16a-b), indicating a brittle fracture mode. In the twinned sample, the crack can be trapped at the TBs for a while. In the period of crack trapped by a TB, plastic deformation delocalizes at the next weak phase far from crack tip, resulting in additional plastic dissipation, as shown in Supplementary Figures 16c-d. By controlling the cohesive stress in the tangential direction, one can observe apparent crack tip blunting and TB sliding. Supplementary Figures 16g and 16h show the stress-strain curves and associated fracture energies from different modeling cases, respectively. It indicates that the introduction of TB sliding can significantly enhance the facture toughness. Some main parameters used in FEM modeling are given as follows,

(a) Twin phase: cohesive stress is set to 3 GPa, yielding stress 1.2 GPa, and fracture energy 1 J/m^2;

(b) Matrix phase: cohesive stress 9 GPa, yielding stress 4 GPa, and fracture energy 1 J/m^2;

(c) Sliding boundary: cohesive stresses in the normal and tangential directions are set to 500 and 1.6-4 GPa, respectively;
(d) Anisotropic elastic constants[13] for aragonite: C_{11}=164.4 GPa, C_{22}=112.0 GPa, C_{33}=59.2 GPa, C_{12}=65.3 GPa, C_{13}=39.0 GPa, C_{23}=48.2 GPa, C_{44}=40.5 GPa, C_{55}=33.9 GPa, and C_{66}=49.0 GPa.

Coarse grained simulations based on a triangular lattice model. A coarse-grained modeling approach was adopted to verify the toughening effect of nanocrack pattern observed in the experiment. In this approach, while the simulations were performed using LAMMPS[4], a coarse-grained triangular lattice model (Supplementary Figure 14a) was employed to describe the elastic deformation of aragonite and brittle failure at a critical value of bond elongation Δr_c. The strain energy stored in each bond before breaking can be expressed as,

$$F_s = \frac{1}{2} k \left(r - r_0\right)^2 \tag{13}$$

where r and r_0 are the deformed and undeformed bond lengths, and k is the bond stiffness. The parameter values used in the coarse-grained model are listed as follows,

$$k = 86.603 \text{ N m}^{-1}, \quad r_0 = 10 \text{ nm}, \quad \Delta r_c = 0.3 \text{ nm}, \tag{14}$$

which corresponds to an effective model of aragonite with Young's modulus of 100 GPa and fracture energy of 2.92 J/m^2.

The coarse-grained simulations considered two representative nanocrack patterns characterized by three geometrical parameters: crack length $2a$, spacing d and period s (Supplementary Figure 14b). To obtain the effective modulus and fracture strength of simulated samples, uniaxial tension is applied along the y direction of homogeneous and nanocrack-patterned square samples through step-wise straining, with stress-strain curves shown in Supplementary Figure 14c.

Coarse-grained samples for crack propagation in a two-dimensional nanocrack-patterned strip had dimensions of $2H$=4 μm and L=8 μm, and initial crack length of c=3 μm. The simulations were conducted using NVE ensemble, with the initial temperature set at 0 K. The applied strain rate is estimated to be about 10 s^{-1} so that the simulation is essentially quasi-static.

Calculation of fracture energy. Based on linear elastic fracture mechanics, the critical e nergy release rate, referred to as the fracture energy, of a crack moving in a plane stress st rip can be calculated from the following expression[14],

$$G = 2H \int_0^{\varepsilon_0} \sigma d\varepsilon , \tag{15}$$

where H is the half width of the strip, and ε_0 is the critical strain at which the crack starts to propagate in the simulated sample, leading to a rapid drop in nominal stress (defined as the total force imposed on the sample divided by the initial cross-section of the sample in

the crack plane). In both atomistic and coarse-grained simulations, almost all samples failed in a brittle manner, and Eq. (15) was used to compute the fracture energy by integrating the stress vs. strain curve. For the nanotwinned aragonite sample, the crack blocking effect of TBs induces a plateau in the stress vs. strain curve (Fig. 4e in the main text), giving rise to much higher fracture energy. In this case, the fracture energy was calculated by integrating the stress vs. strain curve from zero to the critical strain at the end of the stress plateau.

Supplementary References

1. ASTM E1820-11. Standard test method for measurement of fracture toughness. *American Society for Testing and Materials* (2011).
2. ASTM E1290-08e1. Standard test method for crack-tip opening displacement (CTOD) fracture toughness measurement. *American Society for Testing and Materials* (2011).
3. Schwalbe, K.H., Newman Jr., J. C., Shannon Jr., J.L. Fracture mechanics testing on specimens with low constraint—standardization activities within ISO and ASTM. *Eng. Frac. Mech.* **72**, 4, 557-576 (2005).
4. Plimpton, S. Fast parallel algorithms for short-range molecular dynamics. *J. Comp. Phys.* **117**, 1, 1-19 (1995).
5. Xiao, S., Edwards, S. A. & Grater, F. A new transferable force field for simulating the mechanics of CaCO3 crystals. *J. Phys. Chem. C* **115**, 20067-20075 (2011).
6. Gale, J. D. GULP: A computer program for the symmetry-adapted simulation of solids. *J. Chem. Soc, Faraday Trans.* **93**, 629-637 (1997).
7. Massaro, F. R., Bruno, M. & Rubbo, M. Surface structure, morphology and (110) twin of aragonite. *Cryst. Eng. Comm.* **16**, 627-635 (2014).
8. Zhu, B., Xu, X. & Tang, R. Hydration layer structures on calcite facets and their roles in selective adsorptions of biomolecules: A molecular dynamics study. *J. Chem. Phys.* **139**, 234705 (2013).
9. Nosé, S. A unified formulation of the constant temperature molecular dynamics methods. *J. Chem. Phys.* **81**, 511-519 (1984).
10. Hoover, W. G. Canonical dynamics: Equilibrium phase-space distributions. *Phys. Rev. A* **31**, 1695 (1985).
11. Tsai, D. H. The virial theorem and stress calculation in molecular dynamics. *J. Chem. Phys.* **70**, 1375-1382 (1979).
12. Stukowski, A. Visualization and analysis of atomistic simulation data with OVITO-the open visualization tool. *Modelling Simul. Mater. Sci.* **18**, 015012 (2010).
13. Pavese, A., et al. Interatomic potentials for CaCO3 polymorphs (calcite and aragonite), fitted to elastic and vibrational data. *Phys. Chem. Minerals* **19**, 80-87 (1992).
14. Freund, L. B. *Dynamic Fracture Mechanics.* (Cambridge University Press, 1990).
15. Hytch, M. J., Snoeck, E. & Kilaas, R. Quantitative measurement of displacement and strain fields from HREM micrographs. *Ultramicroscopy* **74**, 131-146 (1998).
16. Kearney, C., Zhao, Z., Bruet, B. J. F., Radovitzky, R., Boyce, M. C. & Ortiz, C. Nanoscale anisotropic plastic deformation in single crystal aragonite. *Phys. Rev. Lett.* **96**, 255505 (2006).
17. Evans, A. G. & Fu, Y. Some effects of microcracks on the mechanical properties of brittle solids—II. Microcrack toughening. *Acta Metall.* **33**, 1525-1531 (1985).
18. Hutchinson, J. W. Crack tip shielding by micro-cracking in brittle solids. *Acta Metall.* **35**, 1605-1619 (1987).
19. Ortiz, M. Microcrack coalescence and macroscopic crack growth initiation in brittle solids. *Int. J. Solids Struct.* **24**, 231-250 (1988).
20. Shum, D. K. M. & Hutchinson, J. W. On toughening by microcracks. *Mech. Mater.* **9**, 83-91 (1990).
21. Li, L. & Ortiz, C. Pervasive nanoscale deformation twinning as a catalyst for efficient energy dissipation in a bioceramic armour. *Nature Mater.* **13**, 501-507 (2014).

22. Li, H., Yue, Y., Han, X. & Li, X. Plastic deformation enabled energy dissipation in a bionanowire structured armor. *Nano Lett.* **14**, 2578-2583 (2014).
23. Gao, H., Ji, B., Jäger, I. L., Arzt, E. & Fratzl, P. Materials become insensitive to flaws at nanoscale: lessons from nature. *Procd. Natl. Acad. Sci.* **100**, 5597-5600 (2003).
24. Gao, H. Application of fracture mechanics concepts to hierarchical biomechanics of bone and bone-like materials. *Inter. J. Frac.* **138**, 101-137 (2006).
25. Zhang, Z., Zhang, Y. & Gao, H. On optimal hierarchy of load-bearing biological materials. *Proc. R. Soc. B* **278**, 519-525 (2011).
26. Smith, B. L. et al. Molecular mechanistic origin of the toughness of natural adhesives, fibres and composites. *Nature* **399**, 761-763 (1999).
